# Non-Monotonic Effect of Asphaltenes on Gas Sorption in Polysulfone-Based Nanocomposite Membranes: Molecular Dynamics Simulations Supported by Theoretical Analysis †

**DOI:** 10.3390/polym18202568

**Published:** 2026-10-18

**Authors:** Igor V. Volgin, Leonid I. Klushin, Sergey V. Larin, Sergey V. Lyulin

**Affiliations:** 1 Institute of Chemistry, St. Petersburg State University, 7/9 Universitetskaya Embankment, St. Petersburg 199034, Russia; 2 Department of Physics, American University of Beirut, Riad El-Solh, P.O. Box 11-0236, Beirut 1107 2020, Lebanon

**Keywords:** molecular dynamics, simulations, asphaltenes, polysulfone, solubility, gases, membranes

## Abstract

Asphaltenes represent a potentially useful class of naturally derived carbonaceous nanofillers for modifying the gas-transport properties of polymer membranes. Here, we combine all-atom molecular dynamics simulations with a recently developed analytical theory of free-volume relaxation to investigate the influence of asphaltenes on the transport properties of bisphenol A-based polysulfone and its nanocomposites containing 0–30 wt.% of asphaltenes. The estimated room-temperature density of the nanocomposites decreases with increasing asphaltene content, whereas the mean solubility coefficients of He, O_2_, N_2_, CH_4_, and CO_2_ exhibit a non-monotonic dependence on filler loading, reaching a maximum around 10 wt.% asphaltenes. Relative to neat polysulfone, the mean solubility coefficients of CO_2_, CH_4_, O_2_, N_2_, and He at this loading are higher by approximately 24%, 14%, 10%, 5%, and 3%, respectively. Further increases in asphaltene loading reduce gas solubility relative to this maximum. The He diffusion coefficient also varies non-monotonically, reaching a maximum increase of more than 30% at 10–20 wt.% of asphaltenes. Analysis of the nanocomposite structure, free-volume characteristics, and gas interactions suggests that the solubility trend is primarily associated with changes in fractional free volume accompanying asphaltene aggregation. These results show that moderate asphaltene loading can enhance gas sorption in polysulfone and provide a molecular-level basis for further computational and experimental studies of asphaltene-filled polymer gas-separation membranes.

## 1. Introduction

Membrane-based gas separation is considered one of the most promising alternatives to conventional energy-intensive processes for the separation and purification of gas mixtures [1]. The development of this technology is largely associated with the use of mixed-matrix, or nanocomposite, polymer membranes [2]. Such systems commonly employ specially synthesized structured materials, including zeolites, metal–organic frameworks, and covalent organic frameworks, as fillers [3,4,5]. Alongside these materials of particular interest are inorganic and carbon-containing fillers that can be obtained from natural or industrial feedstocks.

One class of such fillers is asphaltenes, a heavy petroleum fraction conventionally defined as being soluble in aromatic solvents (e.g., toluene) and insoluble in n-alkanes (e.g., n-heptane) [6,7,8]. At the molecular level, asphaltenes are chemically and structurally heterogeneous mixtures of polyaromatic compounds with island- and/or archipelago-type architectures, bearing alkyl substituents, and heteroatom-containing groups. In recent years, asphaltenes have increasingly been considered as functional components of composite materials, including polymer-based systems [9,10,11,12,13,14]. Nevertheless, the effect of asphaltenes on the sorption and gas-transport properties of polymers remains largely unexplored, despite the ability of asphaltenes to sorb gases [15,16]. To the best of our knowledge, the only experimental study elucidating the effect of asphaltenes on polymer gas-transport properties is the work by Wu et al. [17], who investigated HDPE nanocomposites containing 0.5, 1, and 3 wt.% of asphaltenes. The results of this work show that addition of 1 wt.% asphaltenes reduced CO_2_ and O_2_ permeability by 10–15% relative to unfilled HDPE. At the same time further increase in the asphaltene content from 1 to 3 wt.% improved CO_2_ permeability by approximately 10%, while the O_2_ permeability remained nearly unchanged.

Such nonlinear dependence of transport properties on filler content is not unique to asphaltenes and has also been observed in nanocomposites containing other types of fillers [18,19,20,21]. The form of this dependence varied substantially in these systems and could also differ for the sorption and diffusion components of the overall permeability. For example, in polysulfone/SiO_2_ systems, minimum at 5–10 vol.% of SiO_2_ was found for the O_2_, N_2_ and CH_4_ solubility coefficients, despite a monotonic increase in diffusion coefficients with the filler content. At this filler content solubility was lower by 12–16%, whereas at 15–20 vol.% of SiO_2_ it became higher by 6–28% compared to the unfilled polysulfone [18]. Similar behavior was also observed for Matrimid/activated carbon [19], but only for CH_4_, whose solubility coefficient exhibited a minimum at 8 vol.% of the filler being 12% lower than in the unfilled Matrimid. However, a broad maximum at 31 vol.% of the filler was observed for CO_2_ solubility coefficient, which was approximately 16.5% higher than in unfilled polymer. At the same time, diffusion coefficients increased monotonically up to 50 vol.% filler, except for the He with a minimum at 31 vol.% of the filler. For P4VP-co-PnBA/coordination nanocage membranes, CO_2_, CH_4_ and N_2_ solubility coefficients also exhibited an increase at 10 wt.% with simultaneous small minimum of the diffusion coefficient at 5–10 wt.% of the filler [20]. Finally, in Matrimid/ZIF-12, increasing the filler content from 10 to 20 wt.% increased the H_2_, CO_2_, and CH_4_ permeabilities by approximately 75, 124, and 48%, respectively, whereas a further increase in ZIF-12 content to 40 wt.% reduced the permeabilities relative to their maximum values [21]. Taken together, these studies show that the effect of a filler is governed not only by its concentration but also by the balance between the intrinsic sorption capacity of the filler, its dispersion state, the structure of the interfacial region, and filler-induced changes in polymer-chain packing, mobility, and free volume.

For asphaltenes, competition between these factors appears particularly likely because of their chemical heterogeneity and strong tendency to aggregate, the extent of which depends on the polarity of the asphaltene fraction, the chemical nature and viscosity of the polymer, and the preparation method [9,22]. For example, in HDPE, pronounced asphaltene aggregates became visible already at 3–5 wt.% [10]. In contrast, in systems based on a styrene–isoprene–styrene triblock copolymer, micron-scale asphaltene aggregates formed only at 20–40 wt.% [11]. Thus, it can be hypothesized that, in polymers containing aromatic moieties, the balance between asphaltene–asphaltene and asphaltene–polymer interactions may be more favorable for asphaltene dispersion. This, in turn, may create conditions for a beneficial effect of asphaltenes on the transport properties of such systems.

Testing this hypothesis requires simultaneous analysis of the nanocomposites at the molecular level. Molecular dynamics (MD) simulation is one of the most suitable tools for addressing this problem prior to experimental studies. In recent years, MD methods have been widely used to analyze asphaltene aggregation as well as multicomponent systems containing asphaltenes [12,13,14,23,24,25,26,27,28,29,30,31]. Analysis of the studies for pure asphaltenes indicates a gradual increase in model complexity, from individual representative molecular structures to mixtures of model molecules that can account for the molecular polydispersity of the asphaltene fraction [24,25,26,27,28,29,30,31]. At the same time, studies of multicomponent systems containing asphaltenes as fillers often employ a single representative asphaltene structure [12,13,14]. Although this simplification does not reproduce the chemical polydispersity of a real asphaltene fraction, it reduces the number of varying factors, thereby simplifying theoretical investigations.

In the present work, we use all-atom MD simulations to investigate asphaltene-containing nanocomposites based on bisphenol A polysulfone, an aromatic polymer widely considered as a matrix for gas-separation membranes [32,33,34]. The representative island-type A3 molecule was selected as the asphaltene model [26]. We consider systems containing 0, 10.0, 19.9, and 29.2 wt.% asphaltenes and focus on the thermodynamic contribution to the transport properties of He, O_2_, N_2_, CH_4_, and CO_2_. Using our previously developed approach [35], we analyze gas solubility coefficients corresponding to samples obtained by cooling from the melt at experimentally relevant cooling rates. The main objectives of this work are to determine how gas solubility depends on the asphaltene mass fraction, identify the filler content that provides the most pronounced effect on the sorption properties of polysulfone, and establish the molecular origin of the observed changes through a combined analysis of free volume and asphaltene aggregation.

## 2. Materials and Methods

### 2.1. Materials

#### 2.1.1. Polysulfone

**Figure 1:**
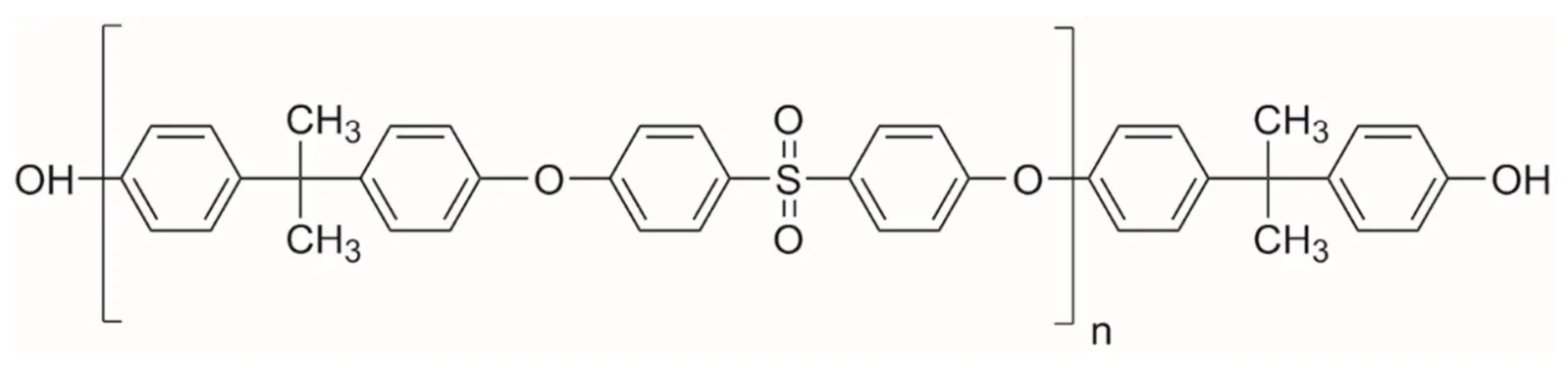
Structure of the polysulfone repeat unit.

Commercial bisphenol A polysulfone [36], Figure 1, was selected as the model polymer matrix for the gas-separation membrane. This amorphous glassy thermoplastic polymer contains rigid aromatic fragments, polar sulfone groups, ether linkages, and methyl groups within the bisphenol A units of its macromolecular chains. This chemical structure contributes to the high thermal, oxidative, and chemical stability of polysulfone, while its amorphous nature and chain architecture also provide good film-forming ability. Moreover, the literature provides substantial experimental data on its transport and structural properties, enabling theoretical results to be compared with previous studies. For this reason, polysulfone was chosen as a suitable model polymer for investigating the effects of asphaltenes on gas-transport properties.

#### 2.1.2. Model Asphaltene Molecule

**Figure 2:**
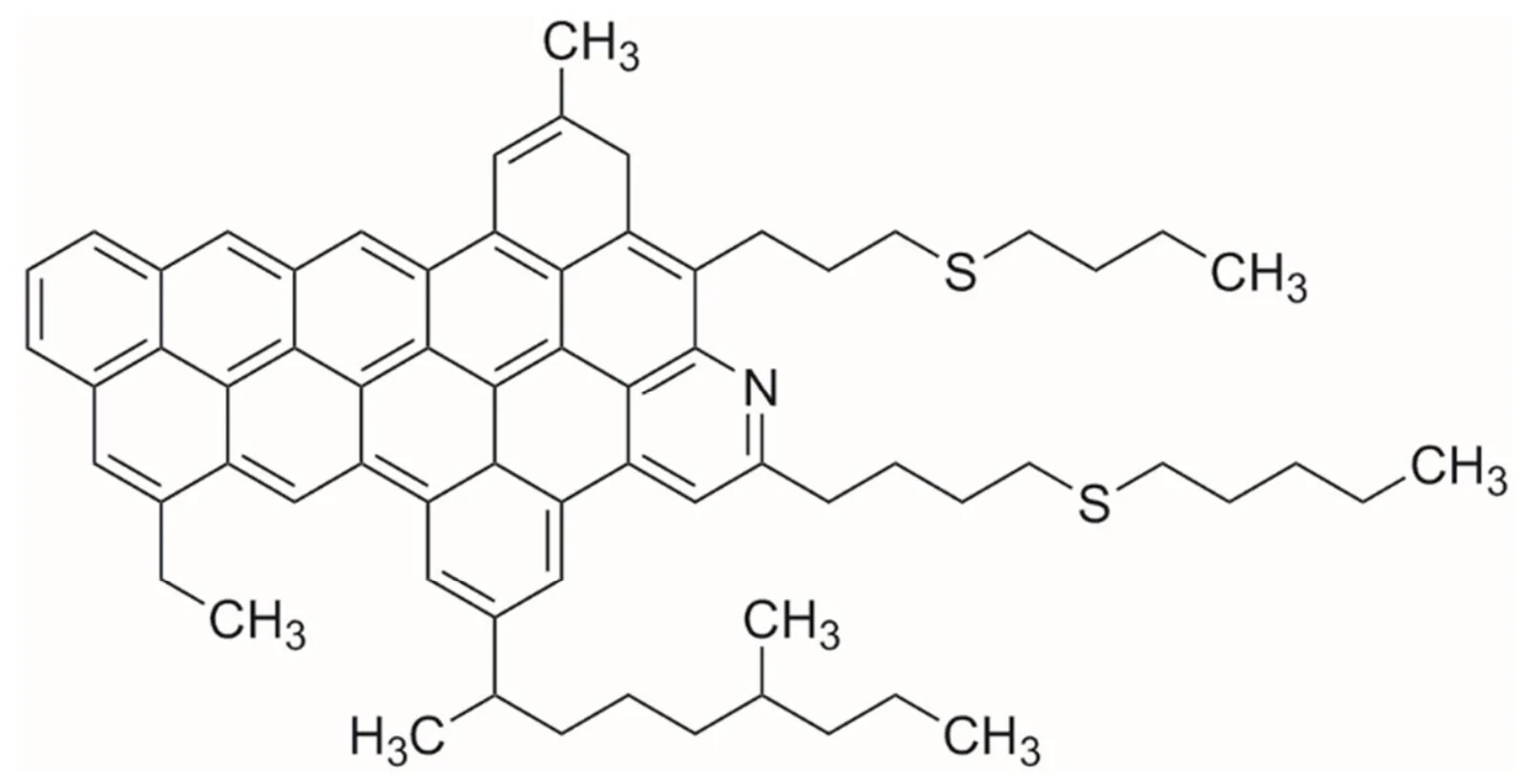
Structure of the model asphaltene molecule [26].

In this work, the A3 structure reported by Pétuya et al. [26] was selected as the model asphaltene molecule, Figure 2. This molecule is a representative structure of one of the groups of model asphaltene molecules identified by unsupervised clustering based on the analysis of molecular descriptors. Importantly, the original library of model asphaltene molecules used in that analysis was constructed on the basis of experimental information on asphaltene composition and structure [25]. The A3 model was also shown to reproduce the expected response to changes in solvent type. For systems containing 100 molecules, the average number of molecules per aggregate increases from 8.3 ± 0.2 in toluene to 28.1 ± 1.7 in n-heptane [26], demonstrating that the model reproduces the expected enhancement of asphaltene association upon transition from an aromatic to a paraffinic solvent. This result does not constitute direct experimental validation of the specific A3 structure; however, it shows that the selected model reproduces the characteristic dependence of asphaltene aggregation behavior on the surrounding medium. Thus, the A3 molecule should be considered an experimentally motivated and statistically representative model of island-type asphaltene molecules.

#### 2.1.3. Systems Composition

In our simulations we consider unfilled polysulfone and polysulfone-based nanocomposites containing different mass fractions of asphaltene molecules, Table 1. The molecular models of polysulfone and its nanocomposites were represented by cubic simulation cells with side lengths ranging from 6.5 to 7.5 nm at 800 K, depending on the composition. Each system contained 20 polysulfone macromolecules with a degree of polymerization *n* = 16, which corresponds to approximately 15 Kuhn segments per chain. Corresponding estimates are given in Section S1 of the Supporting Information File. This degree of polymerization was also chosen to accelerate structural equilibration of the macromolecules in the melt and to reduce the risk of artifacts associated with interactions of chains with their own periodic images.

### 2.2. Methods

#### 2.2.1. All-Atom Models

The GAFF (General AMBER Force Field) [37] was used to develop all-atom models of polysulfone and the model asphaltene molecule. This force field was selected primarily because of its successful use in studies of asphaltene properties [26,38]. At the same time, the polysulfone model was developed and validated within this force field for the first time in the present work. Parameters for bonded and nonbonded interactions were assigned using the ACPYPE software interface (version 2023.10.27) [39]. Partial atomic charges describing the electrostatic interactions were obtained using the AM1-BCC approach, which combines semiempirical AM1 atomic charges with bond charge corrections [40]. 

In this work, we considered He, O_2_, N_2_, CH_4_, and CO_2_ as gas penetrants. Parameters for their all-atom models were taken from the literature [41,42,43,44,45]. These parameters reproduce thermodynamic, structural, and dynamic properties of the modeled gases. Gas–polymer interaction parameters were obtained using the standard GAFF combination rules. This approach is commonly used in MD simulations of transport properties of polymer membranes based on different polymers [35,46,47,48,49,50,51].

#### 2.2.2. Initial Configurations

Initial configurations were generated using a protocol which was successfully applied to different polymer systems [52]. First, partially coiled polymer chains were placed randomly in a cubic simulation cell. The system was then compressed isotropically in several stages (1 ns at 50 bar, 2 ns at 150 bar, and 7 ns at 300 bar), followed by stepwise release of the external pressure (5 ns at 150 bar and 5 ns at 1 bar). These stages were performed at *T* = 800 K, which is approximately 340 K above the experimentally observed glass-transition temperature (*T*_g_ ≈ 458–460 K [53,54]). The use of such a high simulation temperature is common in polymer simulations and is primarily intended to accelerate structural relaxation and facilitate the preparation of equilibrated samples. Although this temperature is within the experimental thermal-degradation range for the polysulfone, thermal decomposition is not described in our simulations. Thus, *T* = 800 K should be regarded as a computationally accelerated, highly mobile, melt-like state used for sample preparation rather than as a temperature intended to reproduce real processing or service conditions. After compression and pressure release, the system was annealed by gradual cooling and heating between *T* = 800 K and *T* = 300 K over 40 ns. Electrostatic interactions were switched off during compression and annealing stages to accelerate the dynamics in the systems. Subsequent simulations were performed with electrostatic interactions switched on.

#### 2.2.3. Simulation Details

Computer simulations were performed using the GROMACS 2023.2 package [55]. Electrostatic interactions were calculated using the particle-mesh Ewald (PME) method [56]. A cutoff radius of 0.9 nm was used for van der Waals and short-range electrostatic interactions, which is a standard value for the GAFF. Bond lengths involving hydrogen atoms were constrained using the P-LINCS algorithm [57]. Analytical long-range dispersion corrections were applied to the energy. The simulations were performed in the NpT ensemble with periodic boundary conditions in all directions. Temperature and pressure were maintained using the v-rescale thermostat [58] and C-rescale barostat [59], with coupling constants *τ*_t_ and *τ*_p_ of 1.0 and 5.0 ps, respectively. All simulations used a 2 fs integration timestep. The main simulation stages were (i) equilibration at *T* = 800 K, (ii) cooling to *T* = 300 K, and (iii) main simulation at *T* = 300 K to obtain trajectories for evaluating gas solubility coefficients and other properties of the studied systems in a glassy state.

At the first stage, the systems were equilibrated for 500 ns at *T* = 800 K and *p* = 1 bar. Three independent configurations were selected from corresponding trajectories after the equilibration stage. A detailed discussion of this step is provided in Section S2 of the Supporting Information. Representative snapshots of the equilibrated systems with different asphaltene contents after equilibration at *T* = 800 K are presented in Section S3 of the Supporting Information. All visualizations were made using OVITO [60].

At the second stage, all considered systems were cooled stepwise with a temperature step Δ*T* = 10 K. Thus, the cooling rate was determined by the simulation time at each cooling step. Five cooling rates were considered, 250 K/ns (1.5 × 10^13^ K/min), 100 K/ns (6 × 10^12^ K/min), 25 K/ns (1.5 × 10^12^ K/min), 10 K/ns (6 × 10^11^ K/min), and 1 K/ns (6 × 10^10^ K/min), corresponding to simulation times of 0.04, 0.1, 0.4, 1, and 10 ns at each cooling step, respectively. The resulting data were used to obtain cooling curves.

At the third stage, each sample was subjected to an additional 10 ns NpT simulation at the final target temperature *T* = 300 K and pressure *p* = 1 bar, with trajectory frames recorded every 5 ps (2000 frames per trajectory). These three independent trajectories for each cooling rate were used to evaluate gas solubility coefficients and other properties of the studied systems.

### 2.3. Calculated Properties

#### 2.3.1. Gas Solubility Coefficients

Solubility coefficients of gases were calculated from the following expression [61]:

(1) $$S=V_{\mathrm{mol}}\frac{1}{\mathrm{RT}}\exp\left(-\frac{\mu_{\mathrm{ex}}}{k_{B}T}\right),$$

where *R* is the universal gas constant, *V*_mol_ is the molar volume of the gas at standard conditions, and *μ*_ex_ is the chemical potential of gas insertion estimated by Widom’s method [62].

All solubility values were reduced to standard temperature and pressure (STP): *T* = 273.15 K and *p* = 1.01325 bar. Simulations were performed using GROMACS 2026.2. The number of trial insertions in each trajectory frame was set to 10^5^ (unless otherwise stated). We previously showed that this value is optimal for obtaining statistically significant estimates of the chemical potential and gas solubility in a polyimide membrane [46].

To extrapolate the calculated gas-solubility values corresponding to those obtained at an experimentally relevant cooling rate at *T* = 300 K (see Section 2.3.2), we used an exponential dependence of solubility on the inverse free-volume fraction 1/*f* [35]:

(2) $$S=A_{S}\exp\left(-\frac{B_{S}}{f}\right),$$

where *A*_S_ and *B*_S_ are fitting constants.

#### 2.3.2. Theoretical Analysis of Cooling Curves

To describe the temperature dependence of the specific volume of the studied systems obtained in simulations at different cooling rates, we used an analytical theory based on Struik’s theoretical approach to describe isothermal aging [63]. A detailed derivation of the equations, justification of the assumptions, and overall validation of this approach are given in our previous work [35], while a brief description is also provided in Section S4 of the Supporting Information. The main input data for our approach are several temperature dependences of the specific volume *v* obtained from three MD cooling curves for 100 K/ns (6 × 10^12^ K/min), 10 K/ns (6 × 10^11^ K/min), and 1 K/ns (6 × 10^10^ K/min). Fitting these curves allows us to estimate parameters required for predicting specific volume *v*(*T*) at any cooling rate, as well as the corresponding density values. In the present work, we use this approach to estimate density of neat polysulfone and its nanocomposites at the experimentally relevant cooling rate (*q* = 0.25 K/s).

## 3. Results

### 3.1. Unfilled Polysulfone Sample

#### 3.1.1. Density, Free-Volume Fraction, and Coefficient of Linear Thermal Expansion

**Figure 3:**
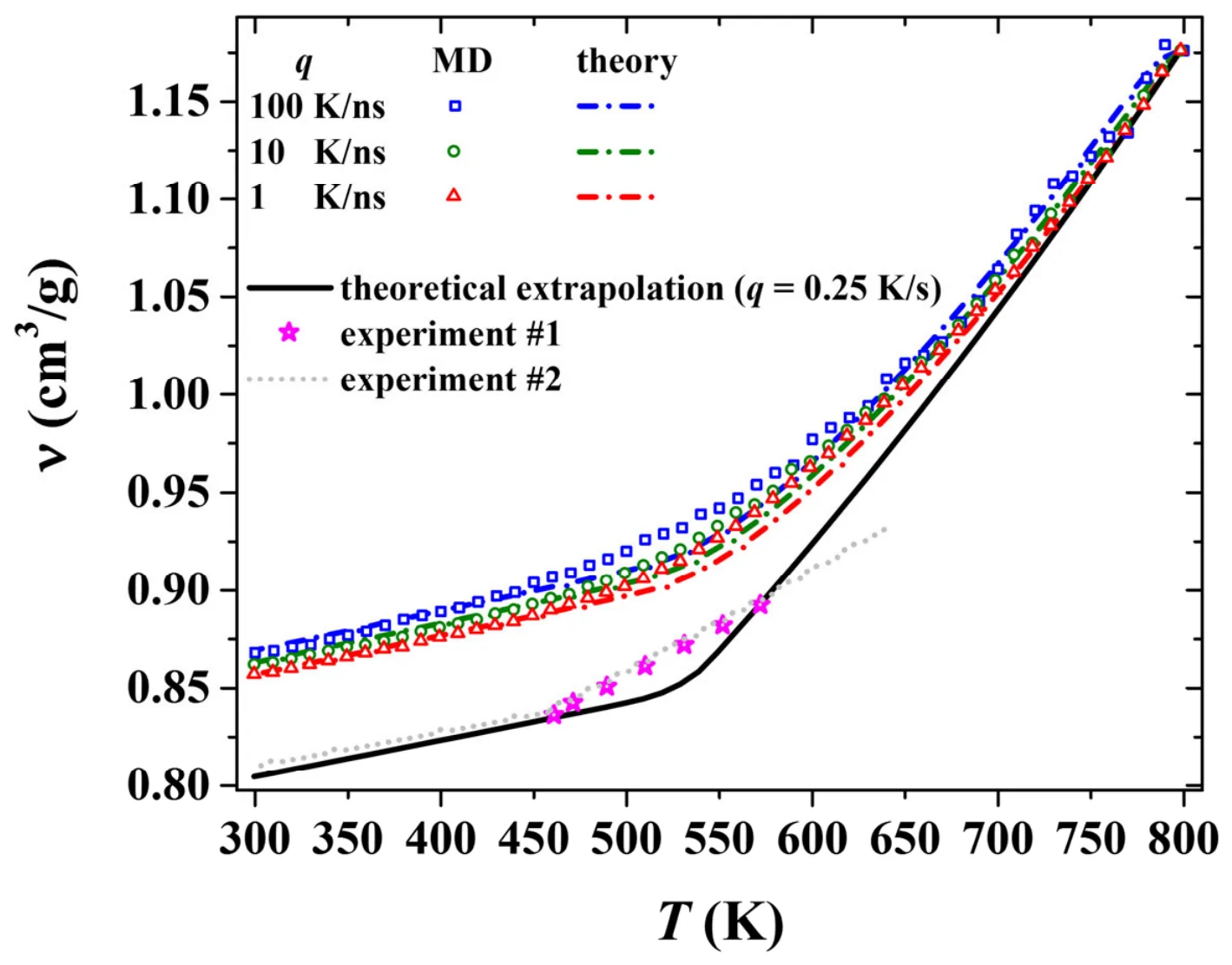
Specific-volume (*v*) cooling curves for different cooling rates (*q*) obtained by MD (open symbols), analytical theory (dash-dotted lines), and theoretical extrapolation to *q* = 0.25 K/s (thick black line). Purple stars and gray dotted line correspond to the experimental data from ref. [53] (experiment #1) and ref. [64] (experiment #2).

At this stage, analytical theory was used to fit the cooling curves obtained from MD simulations for the unfilled polysulfone and to get an extrapolated curve for the experimentally relevant cooling rate (*q* = 0.25 K/s). The results are shown in Figure 3.

Figure 3 shows that the fitted curves reproduce the relative shift in the temperature dependences of specific volume with changing cooling rate, both above and below the apparent *T*_g_ region. The procedure used to determine *T*_g_ and its estimated values are described in Section S5 of the Supporting Information. Figure 3 also shows that theoretical curve for experimental cooling rate (*q* = 0.25 K/s) is in good agreement with the experimental data below 460 K, as well as for the point at *T* = 575 K [53,64]. The deviations observed in the vicinity of *T*_g_ are expected, since the present phenomenological model was intentionally formulated as a simplified extrapolation scheme and was not designed to reproduce the complex relaxation behavior in the glass-transition region. The corresponding extrapolated density and free-volume fraction of unfilled polysulfone are reported in Table 2, together with the coefficient of linear thermal expansion (CLTE) in the glassy state determined during application of the analytical theory.

Table 2 shows that the extrapolated density *ρ* and free-volume fraction *f* of polysulfone, as well as the calculated CLTE values, are close to the experimental ones. Thus, the developed all-atom model of polysulfone provides a reliable basis for further simulations of gas solubility.

#### 3.1.2. Gas Solubility Coefficients

##### Solubility Coefficients Obtained by Extrapolation

**Figure 4:**
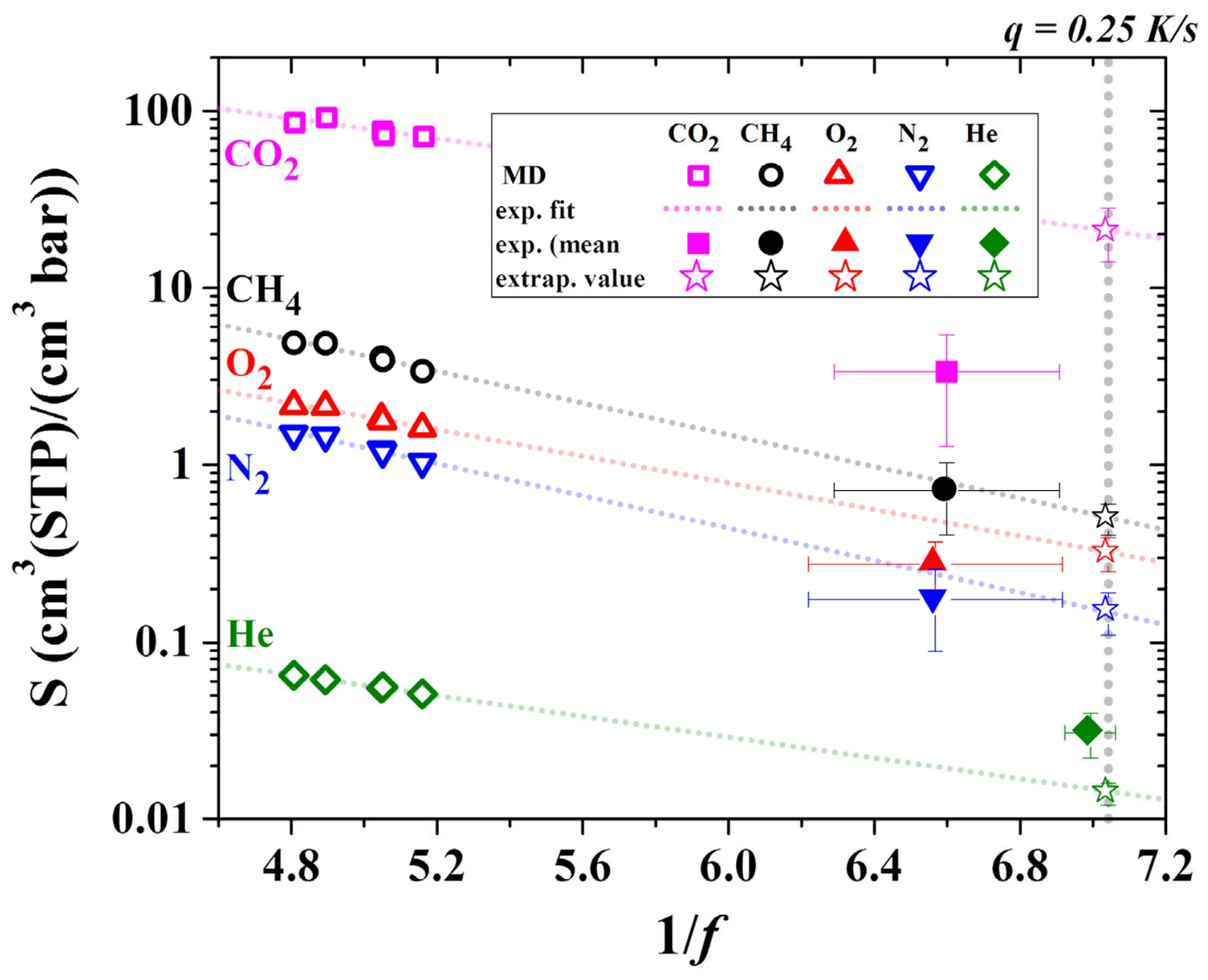
Solubility coefficients (*S*) of gases in the unfilled polysulfone at *T* = 300 K as a function of the inverse free-volume fraction (1/*f*) obtained in simulations (open symbols) and by extrapolating the simulation data (open stars). Averaged experimental values are given by closed symbols.

At this stage, we have estimated solubility coefficients *S* for helium (He), oxygen (O_2_), nitrogen (N_2_), methane (CH_4_), and carbon dioxide (CO_2_) for the unfilled polysulfone. The final values were obtained by extrapolating the simulation data to *f* = 0.142 according to Equation (2). Our results and averaged experimental data (see Table 3 for details) are shown in Figure 4.

**Figure 5:**
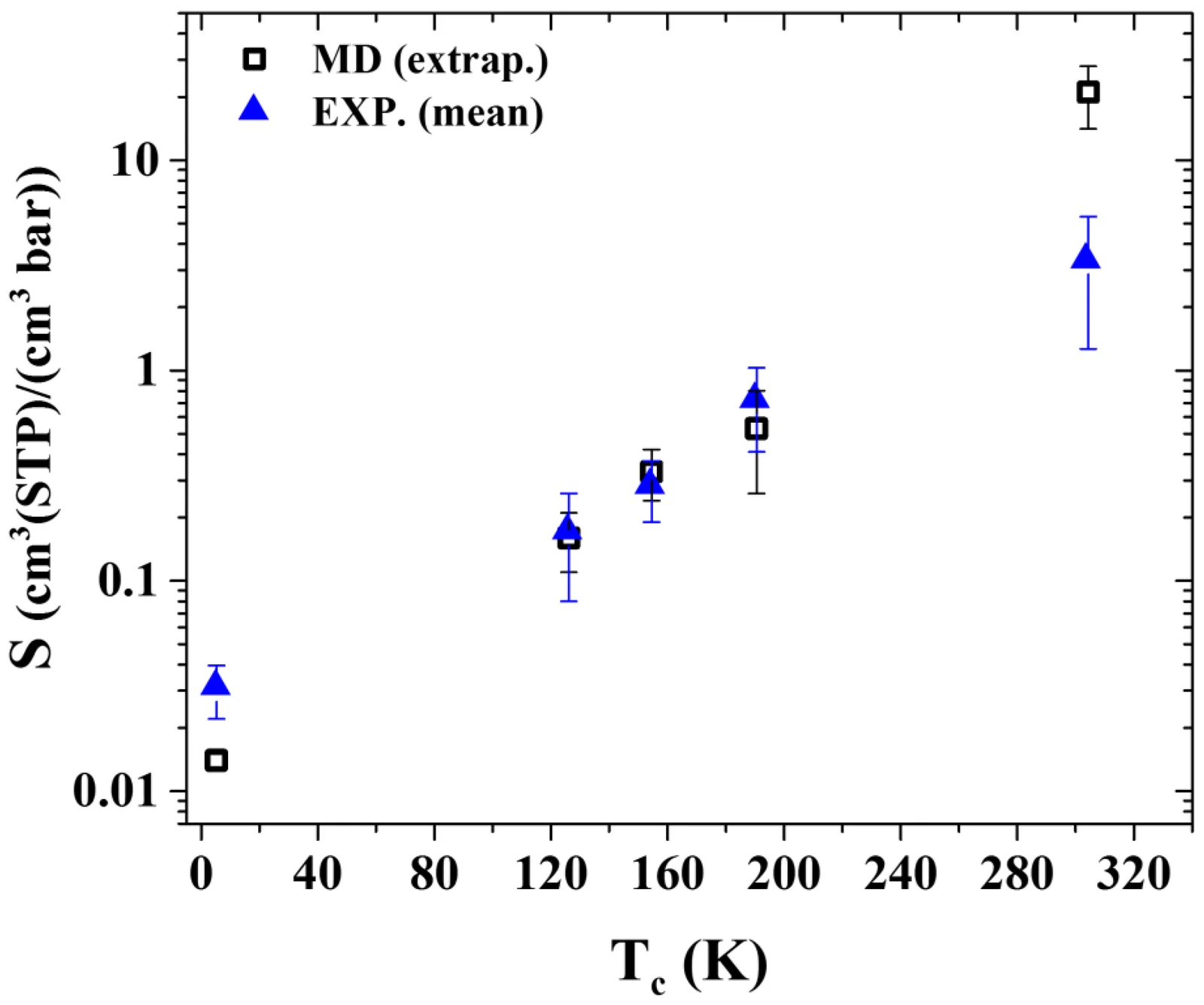
Solubility coefficients (*S*) of gases in unfilled polysulfone as a function of their critical temperatures (*T*_c_), obtained by extrapolating the simulation data (MD(extrap.)) and experiment (EXP.(mean.)).

The data provided in Figure 4 and Table 3 show that solubility coefficients for CH_4_, O_2_, and N_2_ obtained by theoretical extrapolation fall within the range of experimental values and differ only slightly from the mean experimental values. However, for He, the extrapolated solubility coefficient is 2.2 times lower than the mean experimental value, while for CO_2_ we observe almost an order of magnitude difference. To assess the consistency of the obtained solubility values and to identify data points that may be regarded as outliers, we also analyzed the dependence of gas solubility coefficients on critical temperature, Figure 5.

Figure 5 shows that simulation data points for He, O_2_, N_2_, and CH_4_ follow a common *S*(*T*_c_) dependence. In contrast, CO_2_ deviates substantially from this trend, which, in our view, provides evidence that the parameters of the all-atom CO_2_ model require further refinement. In turn, experimental data show good correlation among all the considered gases, while the different slope of *S*(*T*_c_) may result from the following factors: (i) the limited amount of experimental data and its scattering, which complicate the estimation of reliable mean values; (ii) the predominance of experimental measurements performed at a higher temperature (*T* = 308 K); and (iii) fundamental difference between the simulation and experiment approaches used to estimate solubility coefficients. In view of these considerations, we conclude that simulations show reasonable agreement with experiment data for He, O_2_, N_2_, and CH_4_, while results for CO_2_ solubility should be interpreted with caution. Nevertheless, the relative changes in CO_2_ solubility with asphaltene content remain informative.

##### Solubility Coefficients Obtained by Direct Simulation

Although the extrapolated solubility coefficients of He, O_2_, N_2_, and CH_4_ are generally consistent with experimental data, an independent test was performed to calculate them directly for the polysulfone samples at the experimental density predicted by our analytical theory (1245 kg/m^3^ at *T* = 300 K).

To prepare samples at this density, three independent polysulfone configurations obtained after cooling at 100 K/ns (6 × 10^12^ K/min) were isotropically compressed at a rate of 0.001 nm/ps. The compression was adjusted so that their mean density reached 1245 kg/m^3^ at T = 300 K. Note that target density for each sample was selected such that the standard deviation of the mean value mimics that observed in the simulations. After deformation, an additional 10 ns long trajectory was generated for each system at *T* = 300 K to evaluate the solubility coefficients. The simulations were performed in the NVT ensemble to prevent expansion of the simulation cell due to the high internal pressure (3125 ± 219 bar) resulting from compression. Resulting solubility coefficients are reported in Table 4.

The direct solubility estimates for the compressed samples differ from the mean experimental values by factors of 1.5–2.4, depending on the gas (O_2_, N_2_, and CH_4_.), which are larger than corresponding factors 0.7–1.2 for extrapolated solubility coefficients of these gases (see Table 3).

The most likely reason for the quantitative difference between the extrapolated and directly calculated solubility coefficients is the different preparation histories of the materials for which gas solubility was evaluated. The extrapolated values correspond to a material that can be regarded as having undergone relatively slow structural relaxation. By contrast, the solubility coefficients obtained from the direct calculations correspond to a more nonequilibrium sample because it was produced by rapid isotropic deformation. These two preparation histories are expected to result in different distributions, sizes, and connectivity of free-volume elements. For gases interacting less strongly with the polymer (N_2_, O_2_, CH_4_, and He), the differences in these structural characteristics may play a more important role than for CO_2_, whose interactions with the polar groups of polysulfone can make a more pronounced contribution to solubility.

All in all, the alternative procedure described above shows that calculating solubility coefficients of gases in the compressed samples serves as an efficient way to obtain experimentally relevant gas-solubility estimates.

### 3.2. Polysulfone-Based Nanocomposites

Based on the results presented in the previous section, the subsequent analysis of gas solubility in the nanocomposites was performed according to the following steps: (i) estimate the target system density at an experimental cooling rate by extrapolation using the analytical theory [35]; (ii) deform the nanocomposite samples to the different densities, whose values are distributed around the target system density predicted by the analytical theory; (iii) perform simulations for 10 ns in the NVT ensemble at *T* = 300 K; and (iv) calculate gas solubility coefficients of gases.

#### 3.2.1. Evaluation of System Density at Room Temperature

An important pre-requisite for direct evaluation of the solubility coefficients is preparing nanocomposite membrane samples with experimental densities. Since such values are not available for asphaltene-filled polysulfone, we obtained them from the corresponding values of the specific volume at *T* = 300 K for the case of experimentally relevant cooling rate (*q* = 0.25 K/s). Corresponding fitted cooling curves are presented in Section S6 of the Supporting Information File. Theoretically estimated density values for the nanocomposites are given in Table 5.

Having established target density values for the nanocomposite samples, we prepared them using the same procedure as for the unfilled polysulfone. Namely, for each composition, three independent systems obtained at the cooling rate 100 K/ns (6 × 10^12^ K/min) were compressed so that their mean density matched the corresponding target value estimated theoretically. As a result of this compression, the densities were increased by 7.1–8.4% in comparison to the uncompressed samples. However, these systems are also characterized by high internal pressure (see Section S6 of the Supporting Information File). Thus, subsequent trajectories for estimating solubility coefficients were obtained in the NVT ensemble.

#### 3.2.2. Effect of Asphaltenes on Gas Solubility

**Figure 6:**
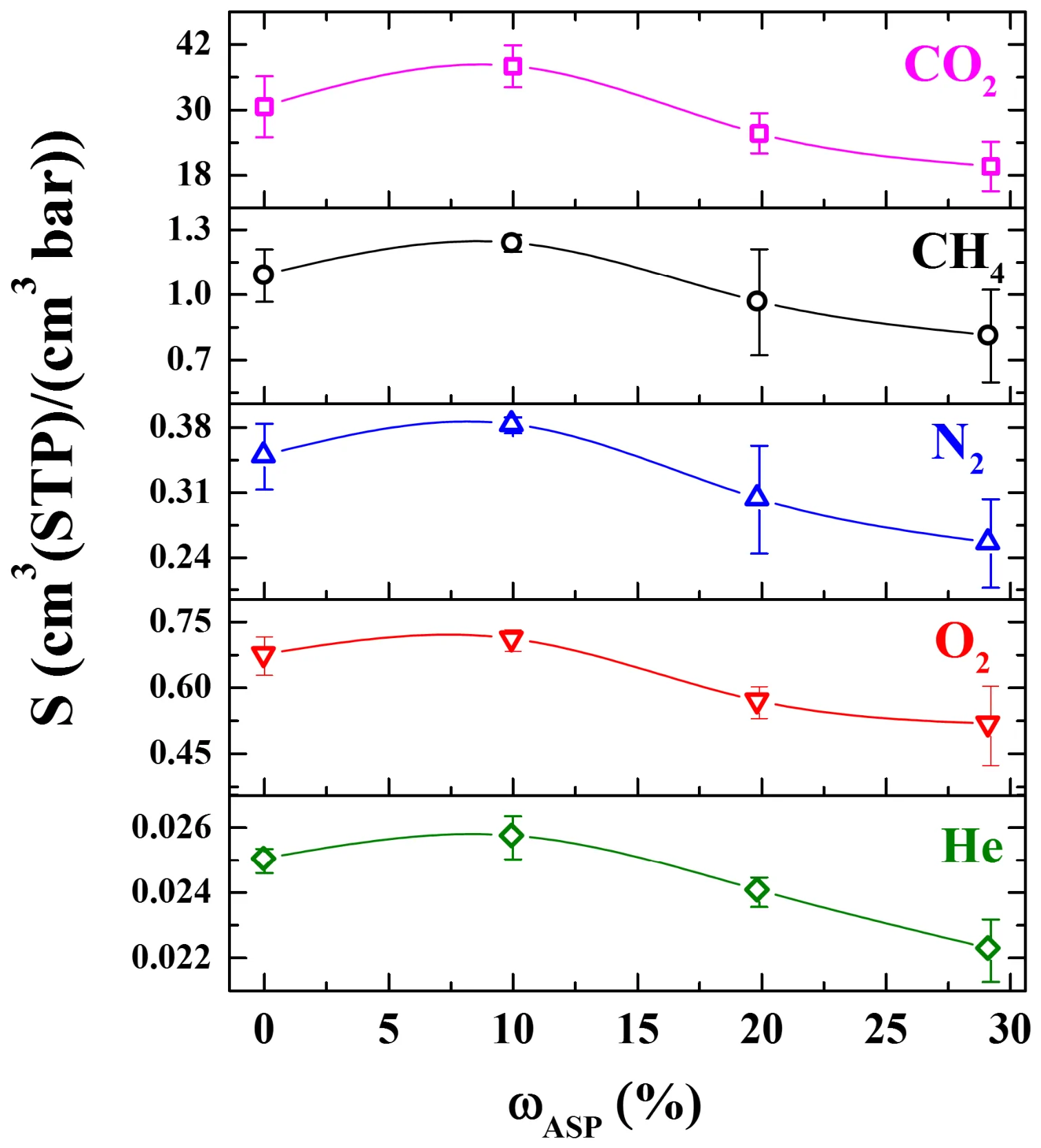
Solubility coefficients *(S)* of gases in the polysulfone at *T* = 300 K obtained in simulations as a function of asphaltene mass fraction (*ω_ASP_*). Lines are guides to the eye.

The calculated gas solubility coefficients for the nanocomposite samples are shown in Figure 6.

For all gases considered, the mean solubility coefficients show a non-monotonic dependence on the asphaltene fraction, with the highest mean values observed around 10.0 wt.%, followed by lower values at 19.9 and 29.2 wt.%. Relative to the unfilled polysulfone, the mean solubility coefficients at 10.0 wt.% asphaltenes are higher by factors of approximately 1.24 for CO_2_, 1.14 for CH_4_, 1.10 for O_2_, 1.05 for N_2_, and 1.03 for He. The largest relative effect is observed for the more condensable gases, whereas He solubility changes only slightly. For CH_4_, CO_2_, and N_2_ at high asphaltene contents, substantial scatter is observed among replicas of the same system. We believe that this primarily reflects heterogeneity among independently prepared nanocomposite structures rather than numerical uncertainty in our calculations. Therefore, the decrease in solubility coefficient from 19.9 to 29.2 wt.% should be interpreted qualitatively. We also repeated our calculations for the configurations obtained by deforming samples after cooling at a lower rate (10 ns/10 K). Similar results were obtained in this case: a maximum near 10.0 wt.% asphaltenes is reproduced for all gases considered (see Section S7 of the Supporting Information).

Another interesting consequence of the observed effect is that asphaltenes modify the solubility selectivity (*α*_S_) differently for different gas pairs (see Section S8 of the Supporting Information). The maximum increase in *α*_S_ relative to unfilled polysulfone is observed around 10 wt.% of asphaltenes and amounts to 9, 14, 18, and 4% for CO_2_/CH_4_, CO_2_/N_2_, CO_2_/O_2_, and CH_4_/N_2_, respectively. In contrast, for O_2_/N_2_, He/CH_4_, and He/N_2_, the maximum occurs at 29.2 wt.% of asphaltenes and amounts to 4, 23, and 23%, respectively.

### 3.3. Origin of the Non-Monotonic Solubility of Gases

#### 3.3.1. Free Volume

Clearly, the observed non-monotonic solubility dependence does not correlate with the previously identified decrease in the mean system density. The most probable factor contributing to this effect is the change in free-volume morphology. To characterize it we calculated the fractional free-volume (*f*) of the investigated systems, Table 6.

At 10.0 wt.% asphaltenes, the fractional free volume *f* is 4% higher than that in pure polysulfone, while further increase in asphaltene mass fraction reduces its amount. Given that comparable changes in fractional free volume are associated with variations in experimentally measured gas solubility coefficients (see Table 3), our results are consistent with the observed non-monotonic dependence of gas solubility on asphaltene content. At the same time, we found no evidence for the formation of additional interactions between gas molecules and the asphaltenes upon their addition (see Section S9 of the Supporting Information File). Taken together, these results suggest a general explanation of the observed effect. At low loading, i.e., up to 10 wt.% asphaltenes, their incorporation into the polymer apparently loosens the sample structure. Further changes in the free-volume properties and, consequently, in gas solubility coefficients are likely associated with asphaltene aggregation.

#### 3.3.2. Asphaltene Aggregation

**Figure 7:**
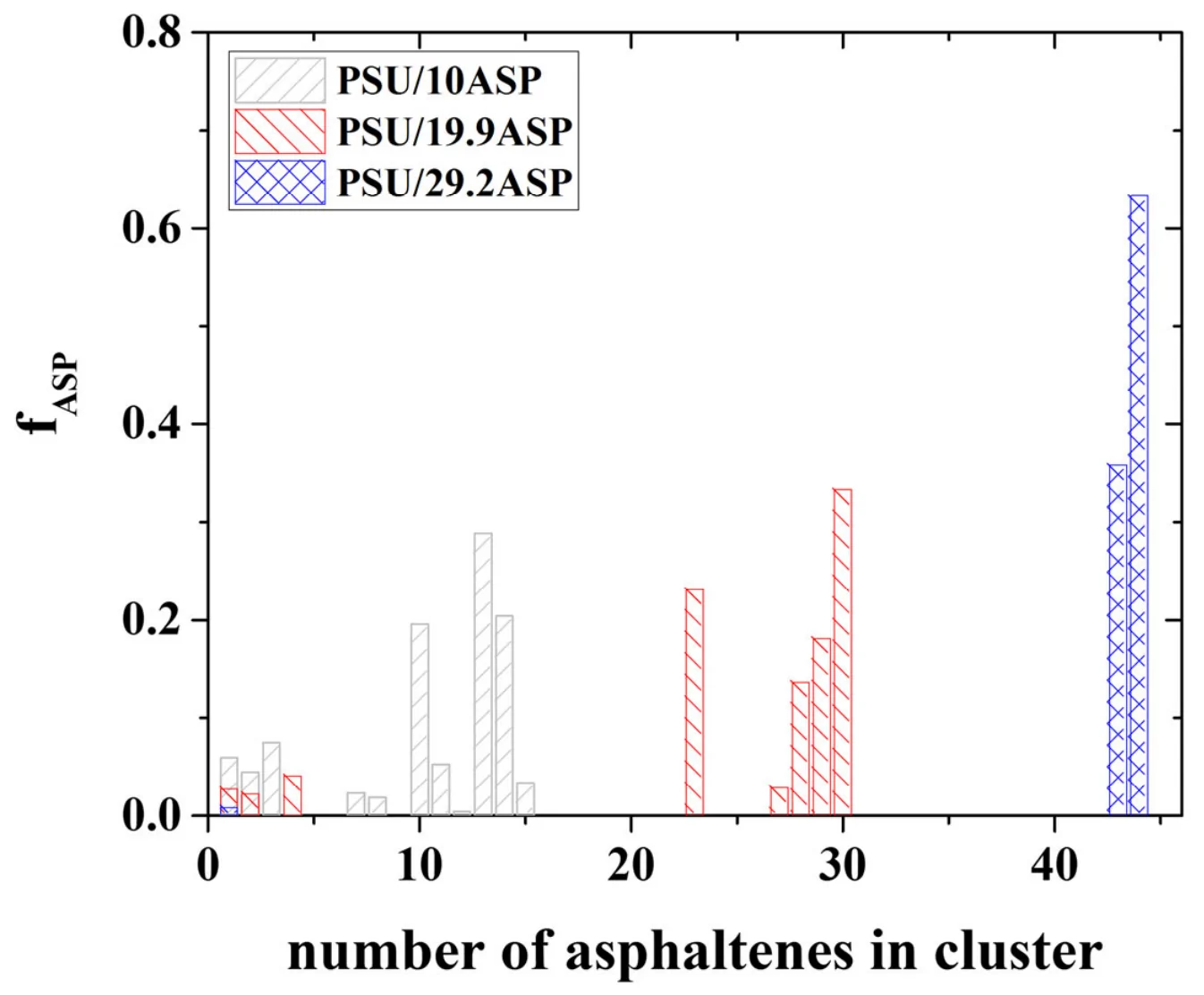
Fraction of asphaltene molecules (*f*_ASP_) belonging to clusters of a given size in polysulfone nanocomposites containing 10.0, 19.9, and 29.2 wt.% of asphaltenes.

To characterize asphaltene aggregation, we calculated the fraction of asphaltene molecules (*f*_ASP_) in the clusters of different sizes. This characteristic was calculated over 200 frames of the 10 ns trajectories using the data provided by the standard GROMACS utility (gmx clustsize) with a distance cutoff *r*_cut_ = 0.4 nm [38]. The results are presented in Figure 7.

**Figure 8:**
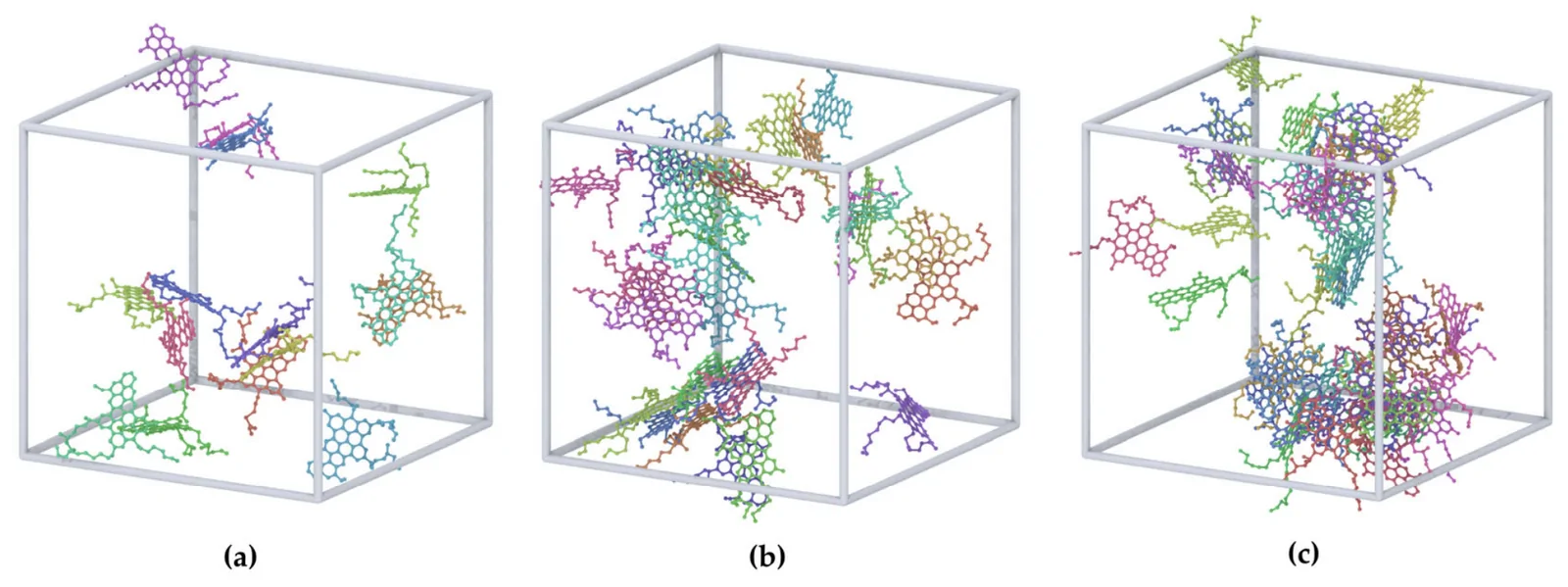
Distribution of asphaltenes in polysulfone nanocomposites containing (**a**) 10.0, (**b**) 19.9, and (**c**) 29.2 wt.% asphaltenes. Hydrogen atoms are omitted for clarity. Individual asphaltene molecules are shown in different colors. Molecules are shown unwrapped across periodic boundaries, and the simulation cell is outlined in gray.

Cluster-size analysis demonstrated that asphaltenes have a strong aggregation tendency in all nanocomposites. The fraction of asphaltene molecules belonging to the clusters composed of more than two molecules exceeded 94% at 10 wt.% asphaltenes and approached 100% at 29.2 wt.%. Moreover, the cluster sizes became larger with increasing asphaltene content, which is also evident from the instantaneous configurations of the simulated systems, Figure 8.

**Figure 9:**
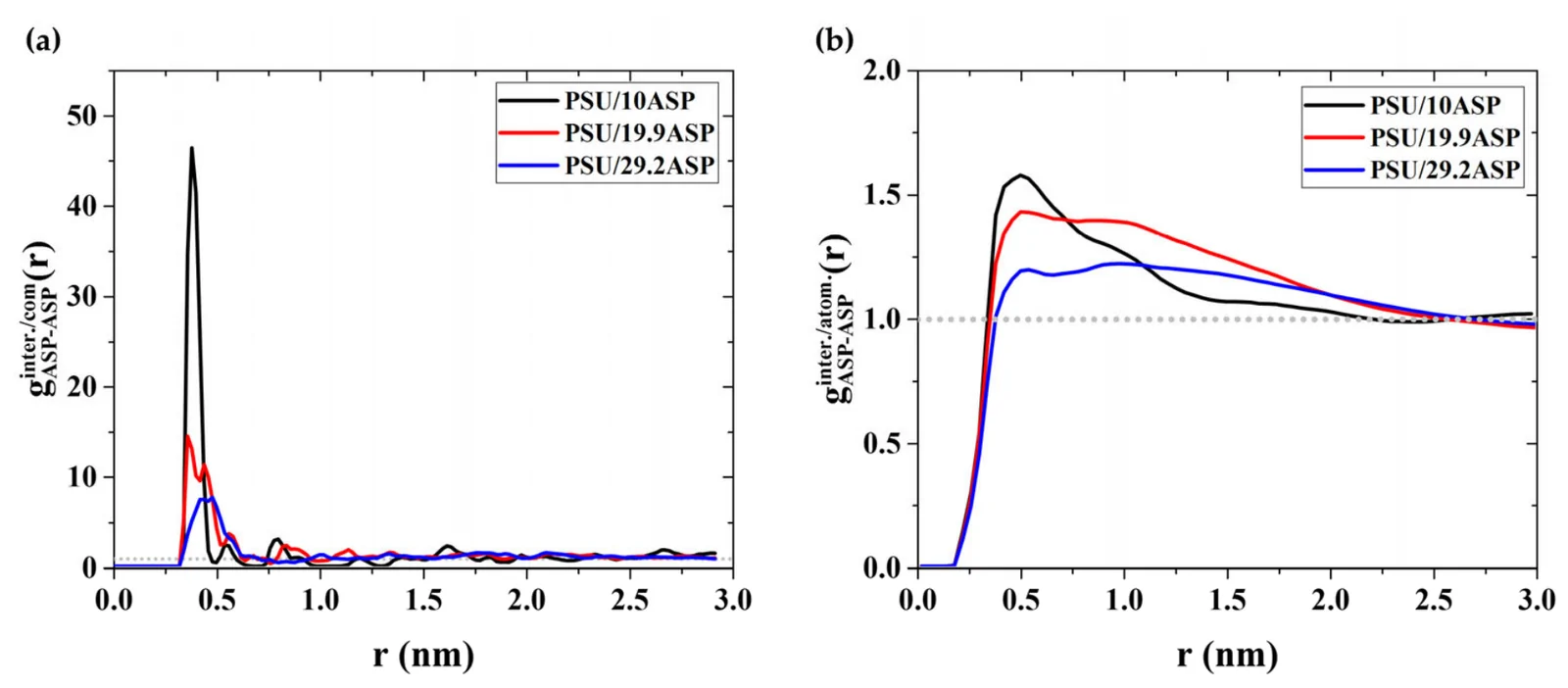
Averaged intermolecular radial distribution functions between (**a**) the centers of mass of the polyaromatic cores and (**b**) all atoms of asphaltene molecules in nanocomposites containing 10.0, 19.9, and 29.2 wt.% of asphaltenes. Gray lines are guides to the eye.

Asphaltene aggregation and local packing were further characterized using intermolecular radial distribution functions calculated for all atoms of the asphaltene molecules, *g_ASP-ASP_*^inter./atom.^(*r*), and for the centers of mass of their polyaromatic cores, *g_ASP-ASP_*^inter./com.^(*r*), Figure 9.

For g_ASP-ASP_^inter./atom.^(*r*), the first maxima are observed at approximately 0.50, 0.50, and 0.54 nm for the systems containing 10.0, 19.9, and 29.2 wt.% of asphaltenes, respectively. Thus, the characteristic distance between the nearest atoms of different asphaltene molecules remains nearly unchanged. At the same time, the height of the first peak decreases, and becomes less pronounced and broader, indicating greater variability in the nearest intermolecular contact distances. However, the decrease in the height of g_ASP-ASP_^inter./atom.^(*r*) should not be interpreted as a marker of decreasing aggregation because the function is normalized by the average number density of asphaltene molecules.

The *g*_ASP-ASP_^inter./com.^(*r*) function provides a more direct measure of the relative arrangement of the asphaltene polyaromatic cores. At 10.0 wt.%, a narrow first peak is located near 0.38 nm, corresponding to a single predominant distance between neighboring cores. At 19.9 wt.%, two closely spaced maxima are observed near 0.36 and 0.44 nm, indicating two preferred mutual arrangements of the asphaltene molecules. At 29.2 wt.%, the main maximum shifts to 0.48 nm, indicating an increase in the most probable distance between neighboring aromatic cores. These characteristic distances are consistent with previous molecular-dynamics studies of asphaltene aggregation [13,26,27,28]. In those studies, distances of approximately 0.35–0.4 nm were associated with more tightly packed parallel aromatic cores in a cofacial, face-to-face stacked geometry, whereas distances of approximately 0.5–0.6 nm were characteristic of a parallel-displaced, offset-stacked geometry. Comparison with these data suggests that more tightly packed parallel pairs of aromatic cores predominate at 10.0 wt.% of asphaltenes, whereas a further increase in asphaltene content leads to the increasing number of configurations with greater mutual displacement of the polyaromatic cores.

## 4. Discussion

### 4.1. Impact of Asphaltenes on Gas Diffusion

**Figure 10:**
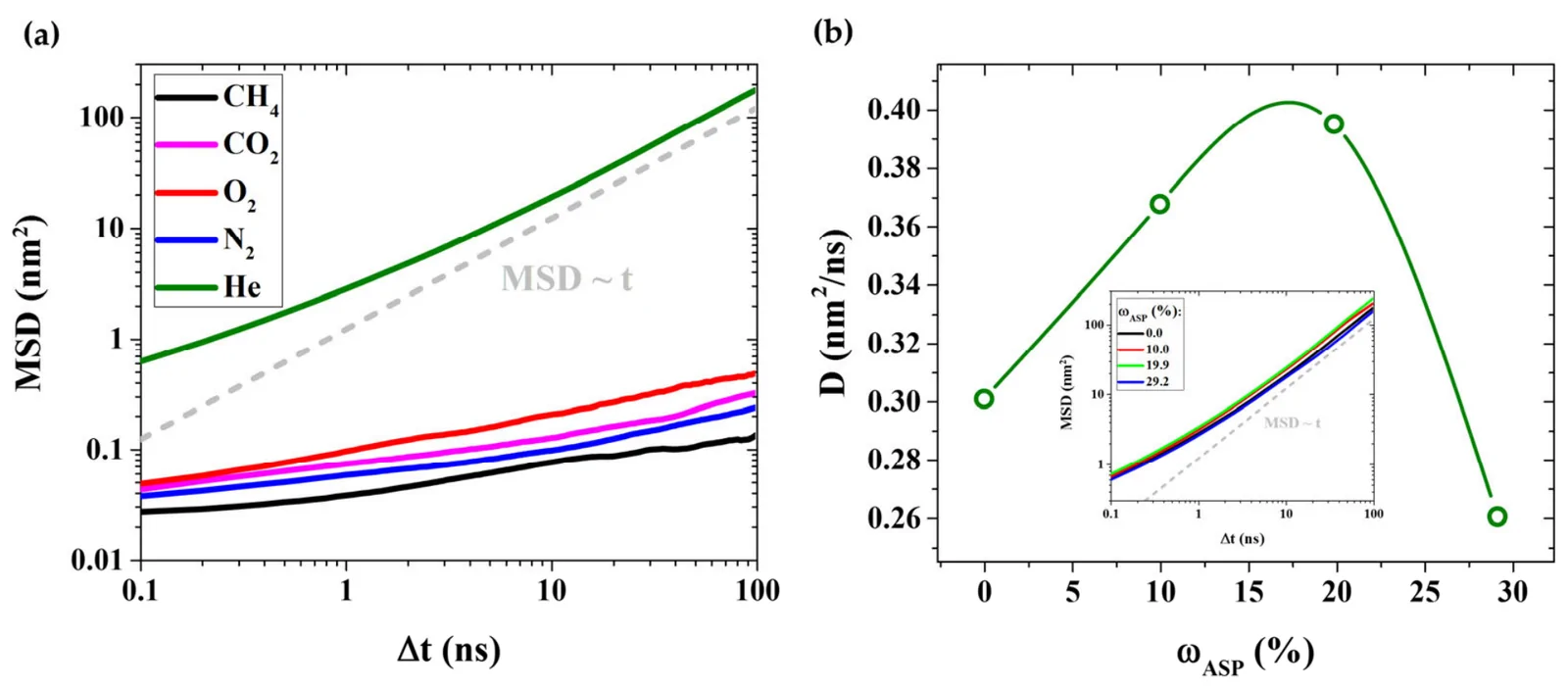
(**a**) Time dependence of the mean-square displacement of gases in unfilled polysulfone at *T* = 300 K. (**b**) Dependence of the helium diffusion coefficient on the asphaltene mass fraction (*ω*_ASP_) in polysulfone at *T* = 300 K. Green line is a guide to the eye. Inset: mean-square displacement of helium molecules in the considered systems.

To evaluate the effect of asphaltenes on gas diffusion, we performed additional simulations. In the first stage, we considered diffusion of 10 molecules of each gas (He, N_2_, O_2_, CH_4_, and CO_2_) in the unfilled polysulfone for one replicate of this system. The simulations were carried out for 1 μs in order to determine the diffusion coefficients from the slope of the mean-square displacement in the normal diffusion regime. The obtained results are presented in Figure 10a.

Analysis of the data presented in Figure 10a shows that the normal diffusion regime was reached only for He. The calculated diffusion coefficient (*D*_MD_ = 0.3 × 10^−5^ cm^2^/s at *T* = 300 K) is in good agreement with literature data (*D*_exp._ = (0.39 ± 0.02) × 10^−5^ cm^2^/s at *T* = 308 K [71]), providing additional validation of the models developed in this work. In the second stage, we simulated the diffusion of 10 helium molecules in nanocomposite samples using one replicate of each nanocomposite system. The normal diffusion regime was also reached for He in these systems. The results are presented in Figure 10b. The diffusion coefficients exhibit a similar non-monotonic dependence on the asphaltene mass fraction, as in the case of solubility. However, an increase of more than 30% in the He diffusion coefficient is observed at approximately around 10–20 wt.% of asphaltenes, which substantially exceeds the effect of asphaltenes on its solubility coefficient. At the same time, the shift in the maximum toward 20 wt.% should not be regarded as conclusive evidence that this filler concentration is optimal for enhancing gas diffusion, since gas diffusion was simulated for only one of the three independently prepared samples at each composition. This result highlights the need for further investigations of the observed non-monotonic effect for other gases.

### 4.2. Limitations and Future Research

Despite the trends identified in this work, their interpretation should take several limitations into account. The first and most fundamental limitation concerns the molecular representation of asphaltenes. The real asphaltene fraction is chemically polydisperse and does not have a single strictly defined molecular structure, whereas the present study employs one representative island-type A3 molecule. As discussed above, this choice is not arbitrary: the structure was identified as a typical representative of one group of model asphaltenes through analysis of a molecular library constructed with consideration of experimental data [25,26]. However, such representativeness should not be considered as an experimental validation of A3. From this perspective, the simulations performed and the results obtained should not be regarded as a complete representation of the real experimental system.

The limitation described above also constrains the generalization of the observed non-monotonic dependence of polysulfone sorption properties on filler. We assume that this dependence may persist for asphaltenes with similar molecular and chemical architectures. However, the use of structures of other types or mixtures of several model molecules may alter the observed behavior. Consequently, the magnitude of the non-monotonic effect may change, and the position of the maximum may shift with concentration. In addition, the non-monotonicity itself may become less pronounced or may even disappear. Testing the robustness of the identified mechanism with respect to the molecular representation of the asphaltene fraction is therefore one of the key directions for future work.

The second limitation concerns the quantitative accuracy of the force fields and gas models used. The most substantial discrepancy with experiment is observed for the CO_2_ solubility coefficient. This indicates that the current parameterization of CO_2_ is suitable primarily for analyzing relative changes between systems. To improve the predictive capability of the simulations for CO_2_, its all-atom model requires additional verification and parameter optimization followed by validation against experimental data for unfilled polysulfone.

The third limitation concerns the statistical reliability of the simulation results. For each composition, three independently prepared samples were considered, and in most cases the calculated solubility values show large scatter among the samples. For this reason, differences between systems with similar compositions cannot always be reliably distinguished from the effects of configurational heterogeneity. Accordingly, the non-monotonic behavior should be regarded as a trend established within the set of compositions examined in this work, while the exact position of the maximum cannot be determined precisely. Addressing this issue will require improved statistical reliability through a larger number of independent initial configurations and a denser set of nanocomposite compositions in the vicinity of the currently identified maximum. This will allow us to estimate more precisely the confidence intervals and the robustness of the observed effect.

In view of the above limitations, further investigations are planned along the following interconnected directions: (i) expanding the set of considered molecular representations of asphaltenes to archipelago structures and mixtures of molecules; (ii) verifying and optimizing the parameters of the gas all-atom models, particularly for CO_2_; (iii) increasing the number of independent nanocomposite samples and the number of asphaltene concentrations considered; (iv) performing longer simulations of N_2_, O_2_, CH_4_, and CO_2_ diffusion to estimate their diffusion coefficients; and (v) extending the analysis to the permeability and selectivity of the nanocomposites.

## 5. Conclusions

In this work, we investigated the effect of asphaltenes on the gas-transport properties of bisphenol A polysulfone. The study addressed two interrelated objectives: to assess the potential of asphaltenes as natural carbonaceous fillers for modifying the properties of polymeric gas-separation materials and to determine whether the polysulfone–asphaltene system exhibits the non-monotonic dependence of gas-transport characteristics on filler content previously observed for other filled polymer systems. Particular attention was paid to the sorption contribution to gas transport. For this purpose, all-atom models of unfilled polysulfone and nanocomposites containing 10.0, 19.9, and 29.2 wt.% asphaltenes were developed. A separate stage of the study was devoted to evaluating the ability of the developed polysulfone model to quantitatively reproduce experimentally measured polymer properties. Using an analytical theory, simulation results obtained at cooling rates accessible to molecular dynamics were extrapolated to a cooling rate relevant to the experimental conditions. The calculated coefficient of thermal expansion of glassy polysulfone, as well as its density and fractional free volume at room temperature, are in good agreement with experimental data. For the solubility coefficients, extrapolation as a function of the inverse fractional free volume was performed to the state corresponding to the predicted room-temperature density. The resulting O_2_, N_2_, and CH_4_ solubility coefficients fall within the range of published experimental data. Satisfactory agreement was also obtained for He, taking into account the limited number of available experimental measurements. The largest discrepancy was found for CO_2_, indicating the need for further optimization of the parameters describing its interaction with the polymer.

A direct approach was used to investigate the nanocomposites: the samples were compressed to the density predicted by the analytical theory, after which their sorption and structural characteristics were calculated directly. Validation of this approach for unfilled polysulfone yielded somewhat higher solubility values than the extrapolation-based estimate. A likely reason is the more pronounced nonequilibrium character of the compressed samples. Nevertheless, this approach was selected for the analysis of the nanocomposites because it allows the structure and sorption properties of the systems to be examined directly at a prescribed density without additional extrapolation. For all gases considered, a non-monotonic dependence of solubility on asphaltene content was obtained. The maximum values are observed at around 10.0 wt.% of the filler, after which solubility decreases. Combined analysis of the radial distribution functions, asphaltene aggregation, and fractional free volume suggests that this behavior is associated with structural reorganization of the nanocomposite. At moderate asphaltene contents, relatively small aggregates are formed, which may disrupt the local packing of polysulfone macromolecules and contribute to an increase in the fractional free volume. As the filler content increases further, more pronounced asphaltene aggregation is accompanied by a decrease in fractional free volume and gas solubility. At the same time, the average interaction energy of gas molecules with the system is nearly independent of asphaltene content. Thus, these results suggest that structural changes in the nanocomposite, rather than changes in the average gas–system interaction energy, are primarily responsible for the observed non-monotonic dependence of gas solubility.

Additional analysis showed that the effect of asphaltenes is not limited to changes in the solubility of individual gases. Because the relative change in solubility differs among gases, solubility selectivity also depends on filler content, and the position of its maximum is determined by the specific gas pair. The highest mean α_S_ values for the CO_2_/CH_4_, CO_2_/N_2_, CO_2_/O_2_, and CH_4_/N_2_ pairs are observed around 10 wt.% of asphaltenes, whereas for O_2_/N_2_ and He-containing pairs the maximum occurs at 29.2 wt.% of asphaltenes. Moreover, He also exhibits a non-monotonic dependence of the diffusion coefficient on asphaltene content, with a maximum increase of more than 30% at approximately 10–20 wt.% of the filler relative to unfilled polysulfone.

Thus, the obtained results show that asphaltenes have the potential to modify the sorption and transport characteristics of polysulfone. This makes them a promising class of natural carbonaceous nanofillers for further investigation in other polymeric gas-separation materials.

## Figures and Tables

**Table 1:** Component composition of the studied systems, including the number of polysulfone chains (*N*_chain_^PSU^), their degree of polymerization (*n*), the number of asphaltene molecules (*N*_ASP_), their mass fraction in the systems (ω_ASP_), and the total number of atoms (*N*_atoms_).

System	*N*_chain_^PSU^	*n*	*N*_ASP_	*ω*_ASP_, %	*N*_atoms_
PSU/0.0ASP	20	16	0	0.0	17 940
PSU/10.0ASP	20	16	15	10.0	20 130
PSU/19.9ASP	20	16	30	19.9	22 320
PSU/29.2ASP	20	16	44	29.2	24 364

**Table 2:** Properties of the unfilled polysulfone: density (*ρ*) and fractional free-volume (*f*) at *T* = 300 K, and the coefficient of linear thermal expansion (CLTE) in the glassy state.

Property	Simulation	Experiment
*ρ*, kg/m^3^	1245 ^1^ (extrap.)	1235 [65], 1241 [66], 1240 [67], 1243 [68], 1240 [69], 1230 [70]
*f*	0.142 ^1^ (extrap.)	0.147 [65], 0.156 [67], 0.158 [68], 0.156 [69], 0.162 [70], 0.144 [71]
CLTE, ×10^−5^ K^−1^	6.7 ± 1.5 ^2^ (MD)	5.6 [53,72], 6.0 [54], 5.3 [54], 5.3 [73]

^1^ Value was determined from the extrapolation curve without uncertainty estimate. ^2^ Standard deviations were calculated from three independent samples.

**Table 3:** Solubility coefficients (*S*) of gases in the unfilled polysulfone obtained in simulations, by extrapolating the simulation data, and in experiments [65,66,67,68,69,70,71].

	*S*, cm^3^(STP)/(cm^3^·bar)								
	Simulation		Experiment						
	Extrap.	Exp. (Mean) ^1^	Ref. [65]	Ref. [66]	Ref. [67]	Ref. [68]	Ref. [69]	Ref. [70]	Ref. [71]
T, K	300	-	308	308	308	303	308	298	308
*f*, %	0.142	0.154 ± 0.007	0.147	-	0.156	0.158	0.156	0.162	0.144
CO_2_	21 ± 7	3.3 ± 2.1	2.08	1.64	4.35	6.5	2.1	-	-
CH_4_	0.5 ± 0.1	0.7 ± 0.3	0.67	0.404	0.71	1.23	0.567	-	-
O_2_	0.32 ± 0.07	0.28 ± 0.09	0.29	0.182	0.24	-	0.24	0.4276	-
N_2_	0.15 ± 0.04	0.17 ± 0.09	0.193	0.039	0.24	-	0.15	0.2475	-
He	0.014 ± 0.002	0.031 ± 0.009	-	0.037	-	-	-	-	0.0243

^1^ Mean values were calculated from all available experimental data.

**Table 4:** Solubility coefficients (*S*) of gases in the unfilled polysulfone obtained from simulations of the compressed samples.

Gas	S, cm^3^(STP)/(cm^3^·bar)
CO_2_	31 ± 5
CH_4_	1.1 ± 0.1
O_2_	0.67 ± 0.04
N_2_	0.35 ± 0.03
He	0.025 ± 0.004

**Table 5:** Theoretical estimates for the density (*ρ*) of the polysulfone nanocomposites at *T* = 300 K as a function of asphaltene mass fraction (*ω*_ASP_).

*ω*_ASP_, %	*ρ*, kg/m^3^
10.0	1224
19.9	1217
29.2	1215

**Table 6:** Fractional free volume (*f*) as a function of asphaltene mass fraction (*ω_ASP_*).

*ω*_ASP_, %	*f*
0.0	0.143 ± 0.001
10.0	0.149 ± 0.002
19.9	0.147 ± 0.001
29.2	0.143 ± 0.002

## Data Availability

The data presented in this study are available upon request from the corresponding author.

## Supplementary Materials

The following supporting information can be downloaded at https://www.mdpi.com/article/10.3390/polym18202568/s1, Figure S1: Dependence of the characteristic ratio CN on the number N of virtual bonds between adjacent selected backbone for the unfilled polysulfone at T = 800 K; Figure S2.1: Time dependence of the average end-to-end distance *H*_ee_, radius of gyration *R*_g_, and Debye ratio (*H*_ee_/*R*_g_)^2^ for polysulfone chains at *T* = 800 K in the unfilled system and nanocomposites with different mass fractions of asphaltenes ; Table S1: Mean values and standard deviations of end-to-end distance *H*_ee_, radius of gyration *R*_g_, and Debye ratio (*H*_ee_/*R*_g_)^2^ for polysulfone chains in the unfilled system and nanocomposites with different mass fractions of asphaltenes at *T* = 800 K; Figure S3: Mean-square displacement of the centers of mass of polysulfone chains at *T* = 800 K in the unfilled system and in nanocomposites with different mass fractions of asphaltenes; Figure S4: Representative snapshots of the polysulfone systems at *T* = 800 K containing 0 wt.%, 10.0 wt.%, 19.9 wt.%, and 29.2 wt.% of asphaltenes; Section S4: Description of the analytical theory; Table S2: Glass-transition temperatures *T*_g_ obtained from the MD simulations and analytical theory at different cooling rates; Figure S5: Specific-volume cooling curves obtained by MD simulations and analytical theory for polysulfone nanocomposites with 10.0 wt.%, 19.9 wt.% and 29.2 wt.% of asphaltenes; Table S3: Internal pressure in polysulfone nanocomposites at *T* = 300 K as a function of asphaltene mass fraction; Figure S6: Solubility coefficients of gases in the unfilled polysulfone and polysulfone-based nanocomposites at *T* = 300 K as a function of asphaltene mass fraction; Figure S7: Solubility selectivity of polysulfone for CO_2_/CH_4_, CO_2_/N_2_, CO_2_/O_2_, CH_4_/N_2_, O_2_/N_2_, He/CH_4_ and He/N_2_ gas pairs as a function of asphaltene mass fraction; Figure S8: Weighted interaction energies of gas molecules with polysulfone and asphaltenes at *T* = 300 K as a function of asphaltene mass fraction. References [74,75,76,77,78,79,80,81,82] are cited in the Supplementary Materials File.
