# Supplementary material for: Non-Monotonic Effect of Asphaltenes on Gas Sorption in Polysulfone-Based Nanocomposite Membranes: Molecular Dynamics Simulations Supported by Theoretical Analysis
Source: Polymers (Basel). 2026 Oct 18;18(20):2568. doi: 10.3390/polym18202568 (PMC13708828; doi:10.3390/polym18202568)
Supplement: Supplementary file 1 [file polymers-18-02568-s001.zip › polymers-4553461-supplementary.pdf]

Supporting Information

to

Non-monotonic Effect of Asphaltenes on Gas Sorption in Polysulfone-based Nanocomposite Membranes: Molecular Dynamics Simulations Supported by Theoretical Analysis

I.V. Volgin[1,*], L.I. Klushin[2], S.V. Larin[1], S.V. Lyulin[1]

[1]*Institute of Chemistry, Saint Petersburg State University, 7/9 Universitetskaya Embankment, Saint Petersburg 199034, Russia*

[2]*Department of Physics, American University of Beirut, P.O.Box 11-0236, Riad El-Solh, Beirut 1107 2020, Lebanon*

**i.v.volgin@gmail.com*

S1. POLYSULFONE CHAIN FLEXIBILITY

To assess the flexibility of polysulfone chains, the backbone was represented by a sequence of chemically defined virtual bonds connecting oxygen atoms, quaternary carbon atoms of the bisphenol-A moiety, and sulfur atoms of the sulfone groups. For a subchain containing N consecutive virtual bonds, the characteristic ratio C_N was calculated as [74,75]:

$$C_N = \langle \frac{R_N^2}{N \cdot l_v^2} \rangle, \quad (1)$$

where R_N^2 is the squared intramolecular distance between selected backbone atoms separated by N consecutive virtual bonds, l_v is the root-mean-square length of the virtual bond (0.585 nm) estimated following the IUPAC recommendation for the characteristic ratio calculation [76], and the angle brackets denote averaging over all corresponding chain segments, chains, and analyzed trajectory frames.

The $C_N(N)$ dependence obtained for the unfilled polysulfone is shown in Figure S1.

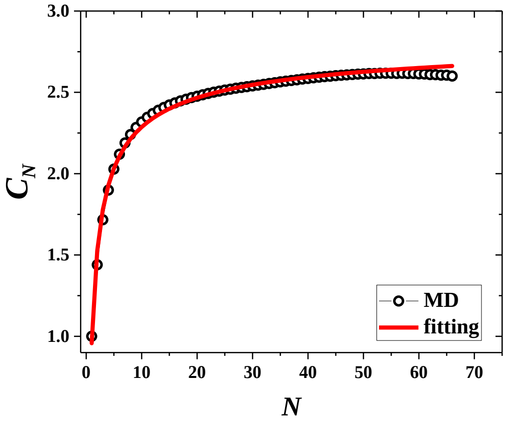

Figure S1. Dependence of the characteristic ratio C_N on the number N of virtual bonds between adjacent selected backbone atoms calculated over the 0-500 ns interval of the main simulation trajectory for the unfilled polysulfone at T = 800 K. Open symbols show the simulation results, while the red line shows the fit according to Equation (2).

To determine the value of the characteristic ratio C_∞ in the limit of large N, we have applied the following fitting function for $C_N(N)$ dependence [77,78]:

$$C_N = C_\infty \cdot (1 - \alpha \cdot N^{-0.5}), \quad (2)$$

where α is the fitting parameter.

Our fit yielded $C_\infty = 2.9$ ($R^2 = 0.995$), which is close to the value $C_\infty = 2.7$ derived from melt-rheology measurements for polysulfone by Herrmann-Schönherr et al. [75]. Using the estimated value of C_∞ we have calculated Kuhn segment length l_K according to the definition of an equivalent freely jointed chain [74]:

$$l_k = \frac{C_\infty N_{all} l_v^2}{R_{max}}, \quad (3)$$

where R_{max} is the estimated contour length of the polysulfone chain, i.e., the maximum end-to-end distance of the fully extended polysulfone chain (31.09 nm), and N_{all} is the total number of virtual bonds (66).

Thus, the calculated Kuhn segment length l_K is 2.11 nm, which is comparable to the l_K = 1.71 nm indirectly estimated by Mei et al. for polysulfone [79]. Within the equivalent freely jointed-chain representation, the simulated polysulfone chain containing 16 repeat units corresponds to approximately 14.7 Kuhn segments.

S2. CHARACTERISTIC DIMENSIONS OF CHAINS AND THEIR MOBILITY

To determine the simulation time required to obtain equilibrated samples of polysulfone and the corresponding nanocomposites, a number of structural characteristics were evaluated: the end-to-end distance H_{ee}, the radius of gyration R_g, and the Debye ratio $(H_{ee}/R_g)^2$. The results are presented in Figure S2.

(a)

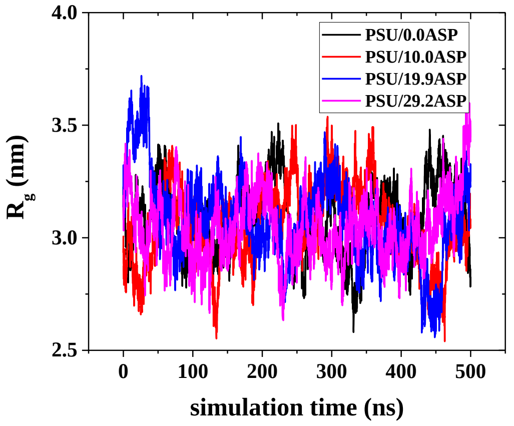

(b)

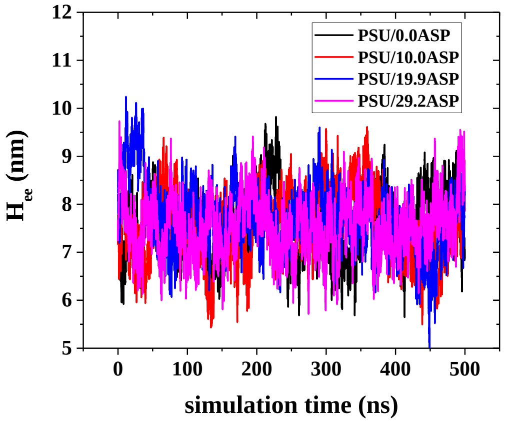

(c)

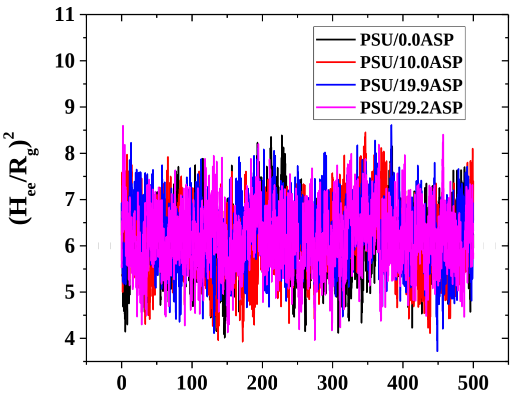

Figure S2. Time dependence of the average (a) end-to-end distance H_{ee}, (b) radius of gyration R_g, and (c) Debye ratio $(H_{ee}/R_g)^2$ for polysulfone chains at T = 800 K in the unfilled system and in nanocomposites with different mass fractions of asphaltenes. The black dotted line corresponds to the value $(H_{ee}/R_g)^2 = 6$, characteristic of equilibrated polymer systems for the case of rather long chains.

Analysis of the data presented in Figure S2 shows that the polysulfone samples can be considered equilibrated after 100 ns of simulation. This is evidenced by the stationary fluctuations in the time series of H_{ee}, R_g, and $(\mathrm{H_{ee}}/\mathrm{R_g})^2$. The average values of H_{ee}, R_g, and $(H_{ee}/R_g)^2$ over the 100-500 ns simulation interval are presented in Table S1.

Table S1. Mean values and standard deviations of end-to-end distance (H_{ee}), radius of gyration (R_g), and Debye ratio $(H_{ee}/R_g)^2$ for polysulfone chains at T = 800 K in the unfilled system and in nanocomposites with different mass fractions of asphaltenes (ω_{ASP}).

ω_{ASP}, %	Characteristic		
	H_{ee}, nm	R_g, nm	$(H_{ee}/R_g)^2$, -
**0.0**	7.6±0.7	3.1±0.2	6.1±0.7
**10.0**	7.6±0.7	3.1±0.2	6.1±0.7
**19.9**	7.6±0.6	3.0±0.2	6.3±0.6
**29.2**	7.5±0.6	3.0±0.1	6.2±0.6

To determine a time interval that would allow independent system configurations to be selected for subsequent cooling, the mean-square displacement $<\Delta r^2_{com}>$ of the centers of mass of polysulfone macromolecules was calculated. The results are presented in Figure S3.

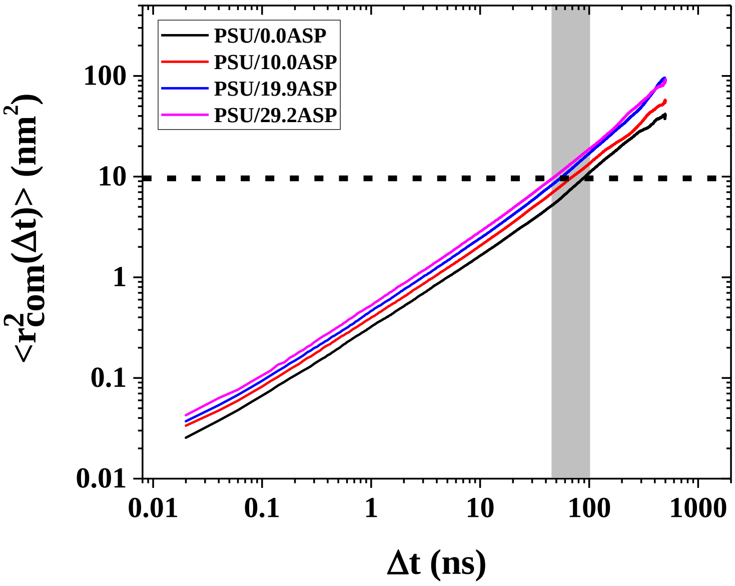

Figure S3. Mean-square displacement of the centers of mass of polysulfone chains at T = 800 K in the unfilled system and in nanocomposites with different mass fractions of asphaltenes. The horizontal dashed line corresponds to the squared radius of gyration R_g^2 of the chains in the unfilled sample (9.6 nm^2). The gray vertical region indicates the characteristic timescale over which the centers of mass of the polysulfone macromolecules undergo a mean-square displacement comparable to R_g^2 = 9.6 nm^2.

Analysis of the data in Figure S3 shows that the mean-square displacement of polysulfone chain centers of mass becomes comparable to the squared radius of gyration of a macromolecule on a timescale of approximately 100 ns. Thus, independent initial system configurations were selected at 100 ns intervals. A total of three initial configurations for each system, corresponding to simulation times of t = 300, 400, and 500 ns, were used for further simulations.

S3. REPRESENTATIVE SNAPSHOTS OF THE SIMULATED SYSTEMS

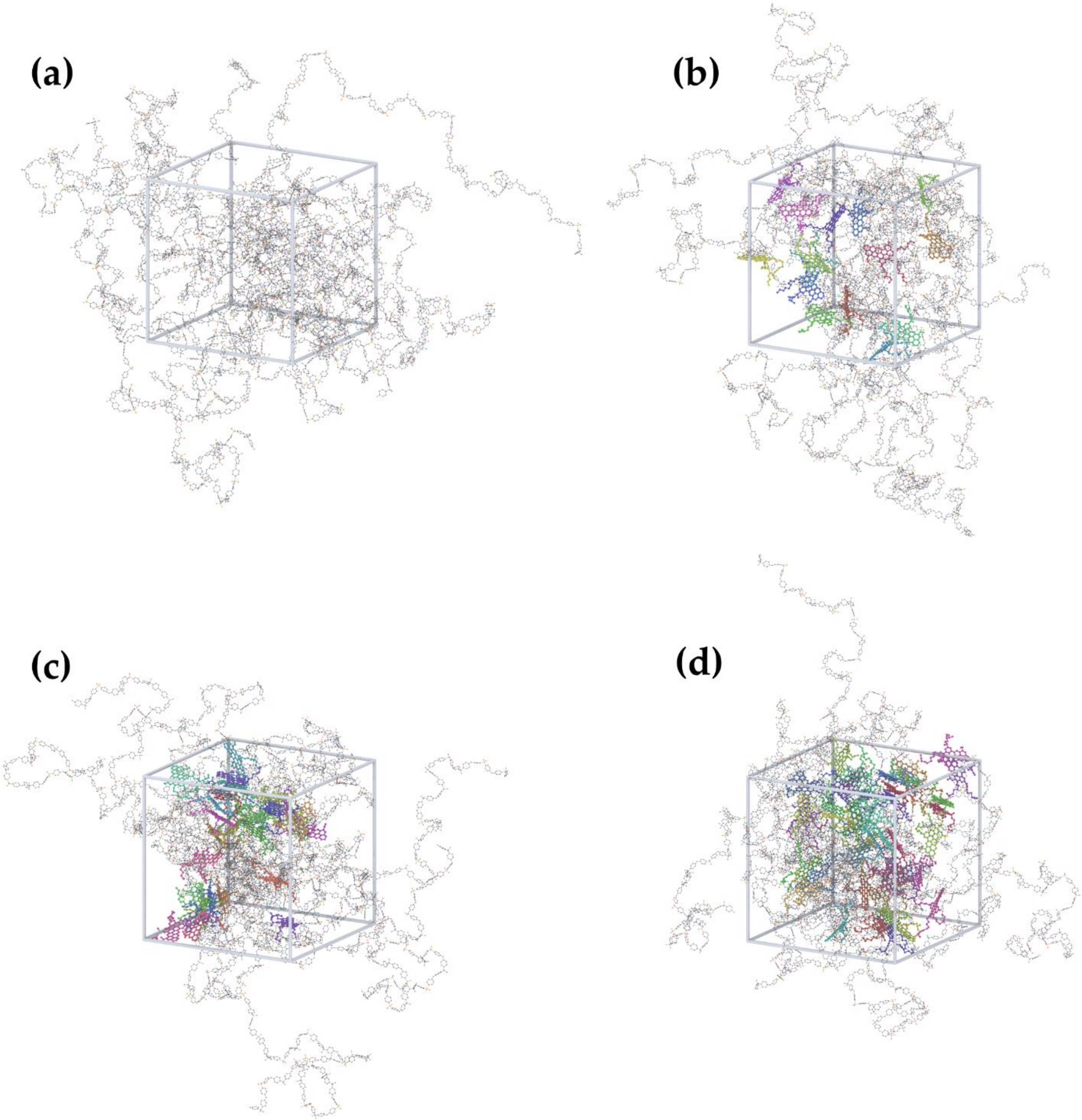

Figure S4. Representative snapshots of the polysulfone systems at T = 800 K containing (a) 0 wt.%, (b) 10.0 wt.%, (c) 19.9 wt.%, and (d) 29.2 wt.% of asphaltenes. Hydrogen atoms are omitted for clarity. Individual asphaltene molecules are shown in different colors. Molecules are unwrapped across periodic boundaries. Simulation cell is outlined in gray.

S4. DESCRIPTION OF THE ANALYTICAL THEORY

Based on the specific volume at each temperature T (and the corresponding time t elapsed since the start of the cooling procedure), the free-volume fraction f was calculated as:

$$f(x) = \frac{v\,(x) - v_0}{v\,(x)}, \tag{1}$$

where x denotes either the temperature T (or the corresponding time t), $v(x)$ is the current specific volume of the polymer at each cooling step, and v_0 is the volume occupied by the atoms of the system, which we estimated by using Bondi's method (i.e., v_0 = 1.3 v_w, where v_w is the van der Waals volume estimated directly from MD simulations with the *gmx freevolume* utility [80] using Bondi's values for the van der Waals radii of atoms [81]).

Because the cooling rate is finite, at each stage of the cooling process the free-volume fraction f at a given temperature T (or time t) increasingly deviates from its equilibrium value f_{eq}, which is determined from Equation (1) by using instead of $v(x)$ the corresponding equilibrium specific volume v_{eq} at temperature T instead of v_0, given by:

$$v_{eq}(T) = v_{eq}(T_0)\exp[\alpha(T - T_0)], \tag{2}$$

where T_0 = 800 K, α is the coefficient of volumetric thermal expansion determined from the high-temperature range (750–800 K) of the cooling curve obtained in the simulation at the lowest cooling rate.

The isothermal relaxation of the deviation of the current free-volume fraction f at each step of the cooling procedure from its equilibrium value f_{eq} was described by Struik's equation:

$$\frac{d}{dt}\left(f(t) - f_{eq}(T)\right) = -\frac{\left(f - f_{eq}\right)}{\tau_\infty(T)e^{-\gamma(f-f_{eq})}}, \tag{3}$$

where γ is a dimensionless parameter characterizing the sensitivity of the relaxation time to deviations of the free-volume fraction from its equilibrium value, and τ_∞ (T) is the near-equilibrium relaxation time described by the Vogel-Fulcher-Tammann equation [82]:

$$\tau_\infty(T) = \tau_0\exp\left(\frac{\delta T_{VF}}{T - T_{VF}}\right), \tag{4}$$

where τ_0 and δ are fitting parameters, and T_{VF}=T_g-53 K.

We assume that the temperature drop causes a rapid affine contraction of the specific volume, $v \to v\cdot\exp(-\alpha_g\,\Delta T)$. The coefficient α_g was determined from the low-temperature range (300–350 K) of the cooling curve obtained at the lowest cooling rate. This temperature range corresponds to the glassy state, where free-volume relaxation is negligible on the simulation timescale.

After the contraction of the specific volume, the following isothermal relaxation in the time interval from t_0 to t was found from an implicit solution of Equation (3) using the exponential integral function *Ei*:

$$Ei\left(-\gamma\left(f(t_0) - f_{eq}(T)\right)\right) - Ei\left(-\gamma\left(f(t) - f_{eq}(T)\right)\right) = \frac{t - t_0}{\tau_\infty(T)}. \tag{5}$$

To avoid nonphysical solutions of Equation (5) at $T \le T_{VF}$, the value of τ_∞ at low temperatures was set equal to its value at T= T_{VF}+5 K, while the parameter γ was assumed to be constant and independent of temperature and cooling rate. The values of the parameters γ, δ and τ_0 were found by simultaneous fitting of the three MD cooling curves for 100 K/ns (6×10^{12} K/min), 10 K/ns (6×10^{11} K/min), and 1 K/ns (6×10^{10} K/min).

S5. DETERMINATION OF T_G FOR INVESTIGATED SYSTEMS

The glass-transition temperature (T_g) at a given cooling rate (γ) was determined by linear fitting of the cooling curves in the low- and high-temperature regions, 300–350 K and 750–800 K, respectively. For the high-temperature region, the same linear function obtained for the slowest simulated cooling rate was used for all cooling rates. The same procedure was applied to the cooling curves predicted by the analytical theory. The resulting T_g values are summarized in Table S2.

Table S2. Glass-transition temperatures (T_g) obtained from the MD simulations and analytical theory at different cooling rates (γ).

**γ**	**T_g, K**	
	**simulations**	**theory**
100 K/ns	621	557
10 K/ns	611	583
1 K/ns	606	606
0.25 K/s	-	567

S6. THEORETICAL ANALYSIS OF NANOCOMPOSITE COOLING CURVES

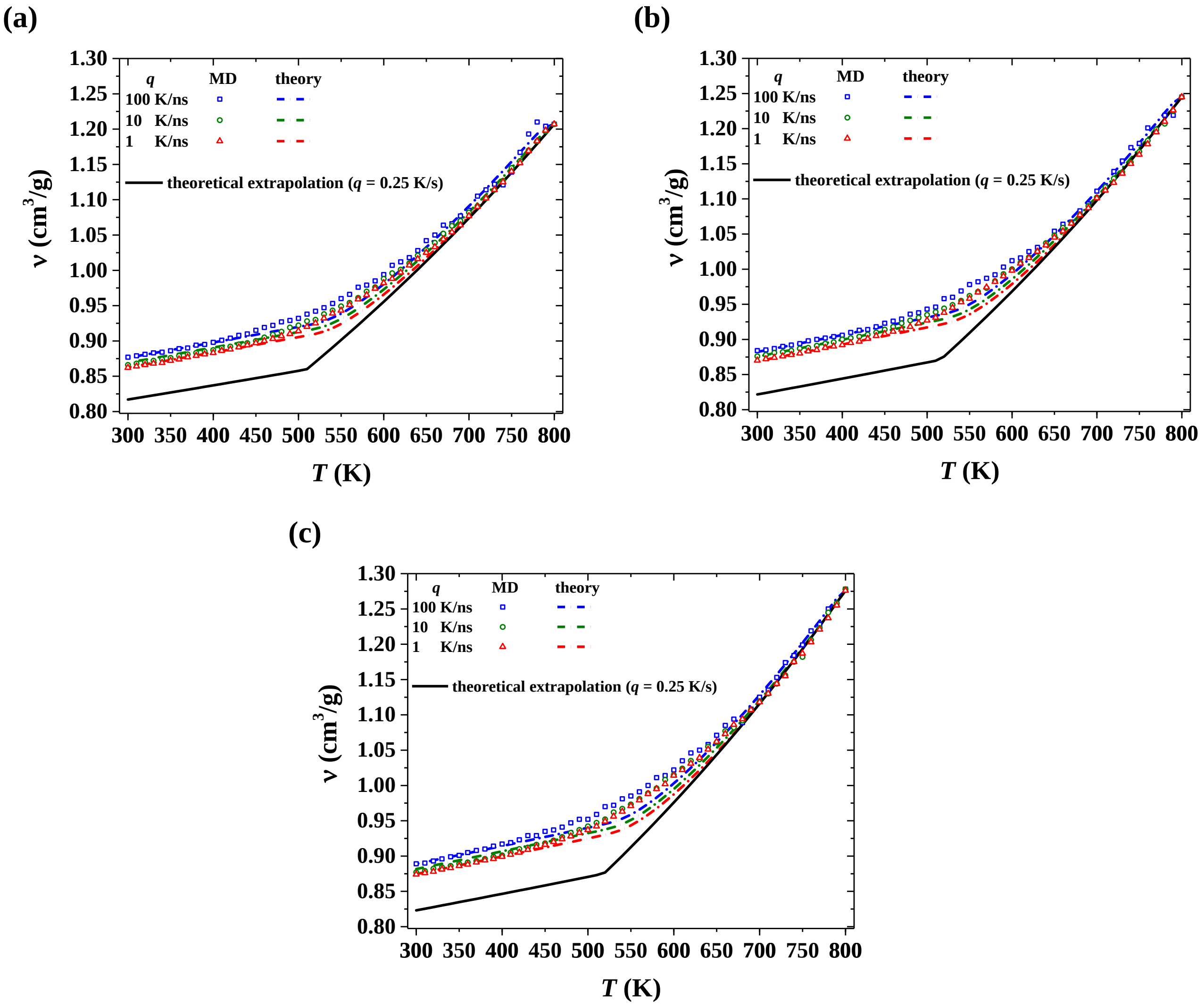

Figure S5. Specific-volume cooling curves obtained by MD simulations (open symbols) and analytical theory (dash-dotted lines) for polysulfone nanocomposites with (a) 10.0 wt.%, (b) 19.9 wt.% and (c) 29.2 wt.% of asphaltenes. The curves are presented for three cooling rates q (shown in the legend), together with the theoretical extrapolation (thick black line) to the experimentally accessible cooling rate q = 0.25 K/s.

Table S3. Internal pressure (p) in polysulfone nanocomposites at T = 300 K as a function of asphaltene mass fraction (ω_{ASP}).

ω_{ASP}, **wt. %**	***p*, *bar***
10.0	2479±203
19.9	2515±164
29.2	2663±149

S7. EFFECT OF ASPHALTENES ON GAS SOLUBILITY (ADDITIONAL RESULTS)

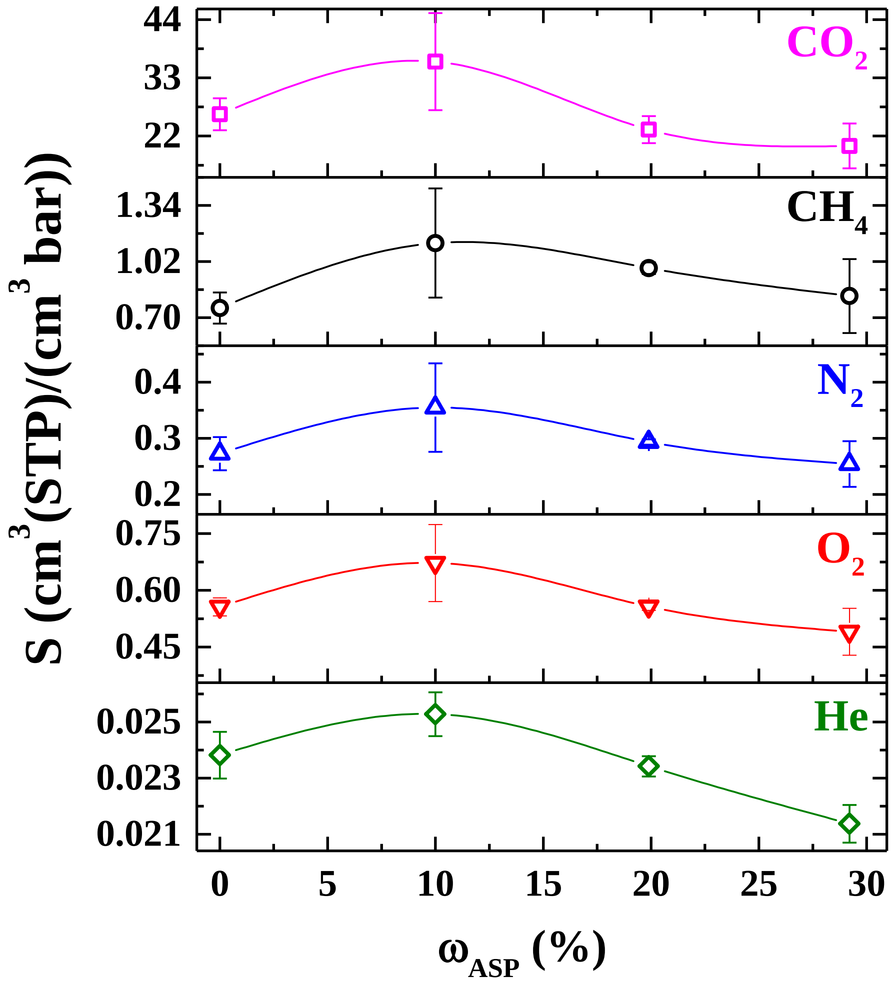

Figure S6. Solubility coefficients (*S*) of gases in the unfilled polysulfone at T = 300 K as a function of asphaltene mass fraction (ω_{ASP}). Lines are guides to the eye.

S8. EFFECT OF ASPHALTENES ON SOLUBILITY SELECTIVITY

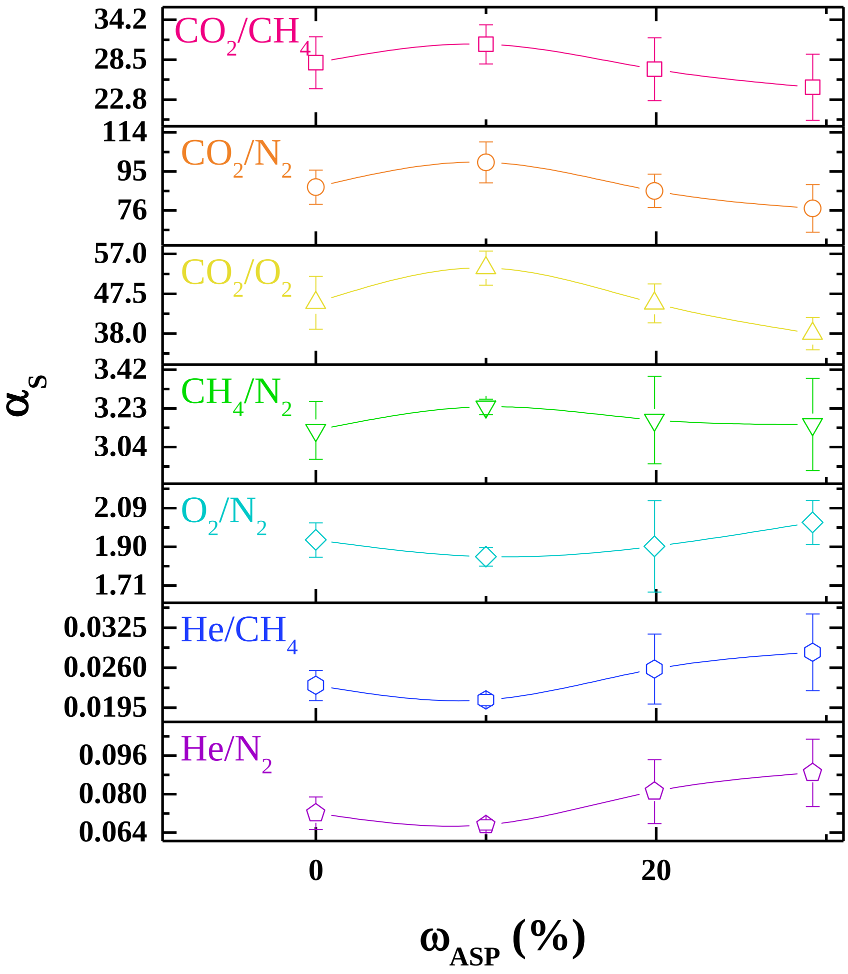

Figure S7. Solubility selectivity (α_S) of polysulfone for CO_2/CH_4, CO_2/N_2, CO_2/O_2, CH_4/N_2, O_2/N_2, He/CH_4 and He/N_2 gas pairs as a function of asphaltene mass fraction (ω_{ASP}). Lines are guides to the eye.

S9. INTERACTIONS OF GASES WITH POLYSULFONE AND ASPHALTENES

To determine the role of gas interactions with the system components, we analyzed the gas interactions with the systems components (polysulfone and asphaltenes) using a specific weighting of the interaction energy. To quantify the interactions of gas molecules with the system components, we calculated the weighted interaction energy:

$$U_{gas-comp.}^{weighted} = \sum_i \mathrm{p_i} \cdot U_{gas-comp.}^{(i)}, \quad (1)$$

where $U_{gas-comp.}^{(i)}$ is the interaction energy of the gas with a given system component (polymer or asphaltenes) upon insertion into the i-*th* free-volume node (see Section 2.3.4 for the definition), and p_i is the normalized insertion weight determined by the total gas–system interaction energy $U_{gas-sys.}^{(i)}$at the i-*th* free-volume node:

$$\mathrm{p_i} = \frac{\exp^{-\beta U_{gas-sys.}^{(i)}}}{\sum_\mathrm{j} \exp^{-\beta U_{gas-sys.}^{(j)}}}, \quad (2)$$

where $U_{gas-sys.}^{(i)}$ and $U_{gas-sys.}^{(j)}$ are the total interaction energies of the gas with the system upon insertion into nodes i and j, respectively, and $\beta = 1/(RT)$.

Each gas molecule was virtually inserted in each of the free volume nodes within 10 random orientations. Corresponding free volume nodes were found using a test spherical particle with the radius r_{probe} = 1.1 Å. Calculations were performed for 200 frames of the 10 ns trajectories using in-house codes.

With this weighting scheme, energetically favorable gas positions contributed more strongly to the average, whereas insertions with severe steric overlap contributed negligibly. The same set of weights was used for polysulfone and asphaltenes, so the sum of the two component contributions reproduced the weighted gas interaction energy with the entire system. The results are shown in Figure S8.

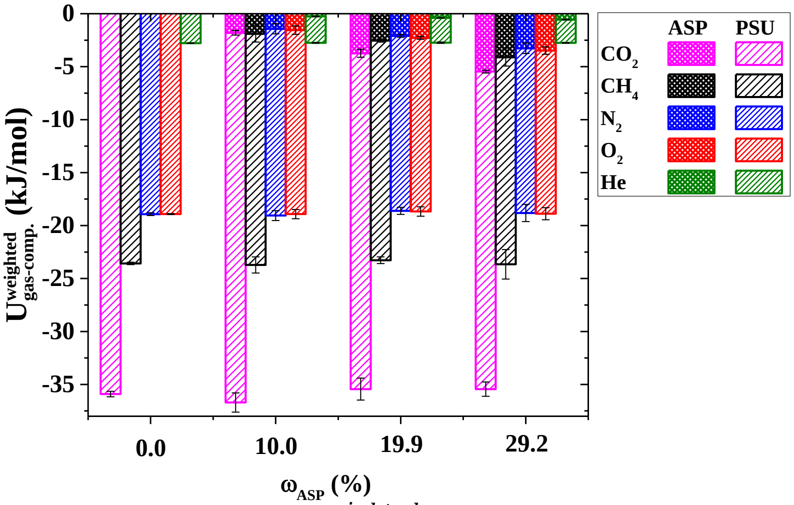

Figure S8. Weighted interaction energy $U_{gas-comp.}^{weighted}$ of gas molecules with polysulfone (filled bars, PSU) and asphaltenes (dashed bars, ASP) at T = 300 K as a function of asphaltene mass fraction (ω_{ASP}). The total bar height corresponds to the sum of the interaction contributions from the two components.

Figure S8 shows that as the asphaltene content increases, their contribution to the gas–system interaction energy becomes stronger, whereas the contribution from interactions with polysulfone becomes weaker. At the same time, the total weighted interaction energy remains nearly unchanged (as indicated by the overall bar heights in Figure S8). Interestingly, over the investigated composition range, the magnitude of the asphaltene contribution increases approximately linearly with asphaltene mass fraction. However, the fraction of the total weighted interaction energy associated with asphaltenes remains systematically lower than their bulk mass fraction. Thus, the compensation between the polysulfone and asphaltene contributions should not be interpreted as evidence of equal interaction energy per unit mass of the two components. Taken together, these results do not explain the non-monotonic dependence of the gas-solubility coefficients on asphaltene content.
